# Functionalized polycarbonates *via* triphenylborane catalyzed polymerization-hydrosilylation†

**DOI:** 10.1039/c9ra05947d

**Published:** 2019-08-23

**Authors:** Kori A. Andrea, Francesca M. Kerton

**Affiliations:** Department of Chemistry, Memorial University of Newfoundland St. John's NL A1B 3X7 Canada fkerton@mun.ca

## Abstract

Triphenylborane catalyzes the copolymerization and terpolymerization of epoxides and CO_2_ to yield polycarbonates with excellent dispersity. *Via* assisted tandem catalysis, these materials could be hydrosilylated in a one-pot fashion yielding modified polymeric materials. Using only a few reagents, materials with glass transition temperatures ranging from 37–110 °C were obtained.

## Abstract 2

Lewis acid-base pair can be used to make functional polymers from carbon dioxide in a single-pot through tandem catalysis.

## Graphical abstract

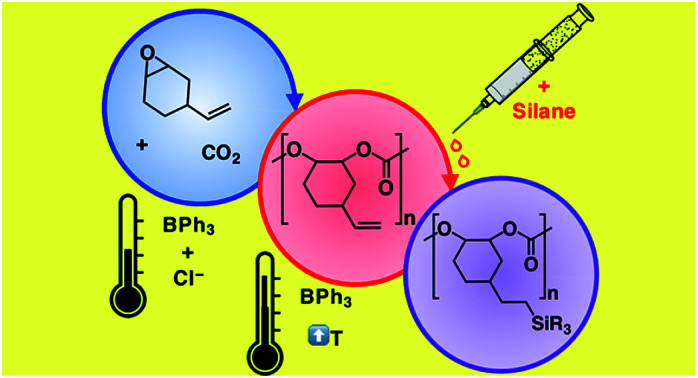

Transformation of carbon dioxide (CO_2_) into useful organic materials is important from an economic and environmental viewpoint.^1,2^ Specifically, the reaction of CO_2_ and epoxides can yield either cyclic carbonates or polycarbonates, with product selectivity relying on several factors such as temperature, pressure, substrate and catalyst design. The polycarbonate product is attractive as it paves a new road towards the development of new sustainable polymeric materials that may serve as alternatives to the traditional petroleum-based products that dominate society today.^3–5^ The use of catalysts that can incorporate a mixture of epoxide monomers into the final product has evolved in recent years, which can allow renewable functional epoxides to be incorporated into a biorenewable end product.^6,7^ Furthermore, such functional epoxides including unsaturated building blocks allow for subsequent modification and tailoring of the polymer and its properties. This has been achieved previously,^8^ for example, *via* olefin metathesis and thiol–ene crosslinking reactions.^8–14^

Copolymerization of epoxides and CO_2_ is usually facilitated by metal-based catalytic systems,^6,15^ but recently the use of organo- and non-metal catalysts has emerged,^16^ including two examples making use of organoboranes. The first used triethylborane to yield polycarbonates with high carbonate content when either propylene oxide (PO) or cyclohexene oxide (CHO) were used as the substrate.^17^ We recently reported the use of arylboranes, both triphenylborane (BPh_3_) and the more Lewis acidic tris(pentafluorophenyl)borane (BCF), as catalysts for the production of either cyclic carbonate or polycarbonate products with substrate dependent selectivity.^18^

Triarylboranes, particularly BCF, either alone or as a Frustrated Lewis Pair (FLP) or within a complex ion-pair are known to catalyze a broad range of reactions,^19–24^ including hydroelementations that possess enormous potential for production of chemicals in a sustainable manner.^25,26^ Specifically, hydrosilylation involves the addition of Si–H groups across C–C, C–O and C–N multiple bonds.^27^ As hydrosilylation of alkenes by BCF had been reported,^21^ along with our recent report of BPh_3_ catalyzed copolymerization of CO_2_ and vinylcyclohexene oxide (VCHO), we were motivated to combine these two reactions in one-pot to yield silylated-polycarbonates. Herein, we report the first example of an alkene hydrosilylation catalyzed by the less Lewis acidic BPh_3_. Building on our previous findings regarding the ability of BPh_3_ to produce perfectly alternating polycarbonates, we report sequential copolymerization-hydrosilylation in a one-pot manner *via* assisted tandem catalysis (Scheme 1). We anticipate that such processes can lead to CO_2_-derived polymers with tailorable physical properties including glass transition temperatures. Also, these polymers may show enhanced solubility in organic solvents, which will facilitate film-casting, and if some Si–H bonds remain, it may allow polymers to be attached to surfaces *via* covalent bonding or grafted to other macromolecular species to form more complex architectures.

**Scheme 1:**
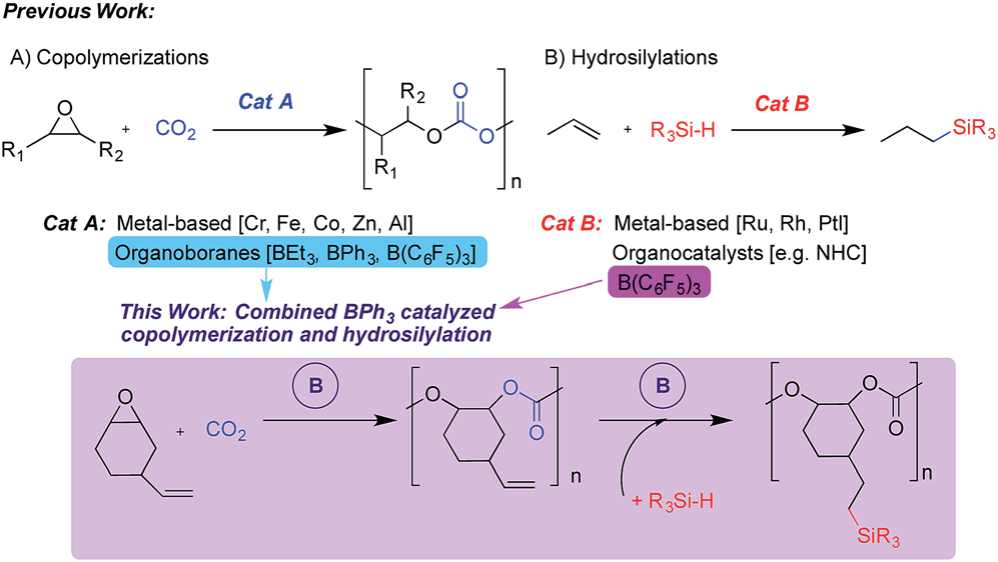
Catalytic copolymerization and hydrosilylation.

The vinyl groups of the polyvinylcyclohexene carbonate (PVCHC) provide several potential routes to polymer modification, which could result in tuning of its physical and chemical properties. This has been done previously using methods such as thiol–ene click chemistry^28^ and metathesis.^10,18^ BCF is known to activate Si–H bonds and facilitate their addition across unsaturated substrates,^21,29,30^ whereas BPh_3_ has been studied to a lesser extent. We envisioned that BPh_3_ would be able to catalyze the addition of Si–H groups onto a vinyl-substituted polycarbonate. Therefore, we performed the following ‘one-pot’ sequence (Fig. 1): BPh_3_ was used to catalyze the copolymerization of VCHO and CO_2_, the CO_2_ was vented and phenyldimethylsilane added to the reaction mixture so the BPh_3_ present could then catalyze the hydrosilylation of the alkene within the polycarbonate. We did not attempt to perform the hydrosilylation reaction in the presence of CO_2_, or prior to or during the copolymerization, as BPh_3_ is able to hydrosilylate CO_2_ but does not react with propylene carbonate,^31^ and we presume other carbonates. We monitored the one-pot process *via in situ* IR spectroscopy. The formation of PVCHC was monitored *via* growth of the carbonate stretch at 1747 cm^−1^. We observed no induction period and signal saturation occurred within approximately 1 h. After 24 h, we cooled and depressurized the vessel before injecting a mixture of phenyldimethylsilane in dichloromethane and heating to 40 °C. A trial hydrosilylation reaction (NMR scale) on isolated PVCHC was successful at this temperature. However, for the one pot process after 4 days, no new bands were observed in the IR spectrum. Upon increasing the temperature to 60 °C, within hours we saw a decrease in intensity of bands at 2122 and 882 cm^−1^ (PhMe_2_SiH), and an increase in intensity of bands at 834 and 791 cm^−1^ demonstrating the successful addition of the silane across the alkene of the polycarbonate. The higher temperature for the one-pot process is likely needed to displace the Cl^−^ anion from the boron centre and allow activation of the Si–H bond by the borane. Cl^−^ is used as a co-catalyst in the CO_2_ epoxide copolymerization process and the NMR scale trial reaction was performed in the absence of PPNCl. The successful one-pot reaction was confirmed with ^1^H, ^13^C, HSQC and refocused INEPT ^29^Si NMR spectroscopy (Fig. S1–S6†), and integration of ^1^H NMR signals for the residual vinyl protons and the silyl protons (Si–(CH_3_)_2_, Si–ArH) showed 10% of the vinyl groups had been modified. In the refocused INEPT ^29^Si NMR spectrum of the product, a new signal appeared at *δ* = −1.26 ppm characteristic of a Si–C saturated bond *cf.* PhMe_2_SiH *δ* = −17.27 ppm. Gel permeation chromatography (GPC) traces show an increase in *M*_n_ for the product, while calculated Mark–Houwink–Sakurada (MHS) confirmation plots show an increased degree of branching in the product (*a* = 0.697 in PVCHC *vs.* silyl-PVCHC *a* = 0.479), further confirming successful functionalization (Fig. S7 and S8†).^32^ As anticipated the silyl-modified polymer exhibited a lower glass transition temperature (*T*_g_) 71.5 °C compared with PVCHC, 99.0 °C. This *T*_g_ may possibly be further decreased if a larger proportion of vinyl groups are converted or a different silane employed. Polycarbonates with relatively low *T*_g_ includes commercially available polypropylene carbonate that finds applications in films and coatings. This one-pot copolymerization-silylation process is an example of assisted tandem catalysis,^33^ as the silane reagent triggers the mode of catalysis to change, and represents a new approach to functionalized polycarbonate.

**Fig. 1:**
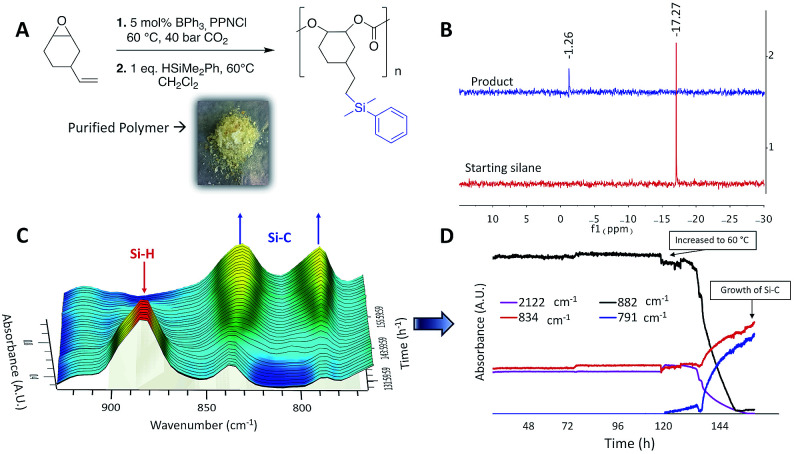
One-pot assisted tandem catalysis to yield silane modified polycarbonates. (A) One-pot catalytic formation of silylated-PVCHC. (B) Refocused INEPT ^29^Si NMR spectra of PhMe_2_SiH and product. (C) *In situ* IR spectra of reaction mixture over time. (D) Reaction profile obtained from *in situ* IR data.

To obtain more examples of functional polycarbonates, while building upon past examples of mixed epoxide/CO_2_ terpolymerizations,^34–38^ we sought to investigate our BPh_3_/PPNCl catalytic system for similar activity. In our previous research, when propylene oxide (PO) was used as the substrate neither polypropylene oxide nor polypropylene carbonate (PPC) was formed.^18^ However, when an initial monomer mixture of 50 : 50 CHO : PO was used, we saw an incorporation ratio of 4 : 1 CHO : PO in the resulting polycarbonate *i.e.* 20% PPC linkages (Table 1, entry 1). Moving to a 10 : 90 CHO : PO monomer mixture, a terpolymer with 50% PPC linkages and a lower *T*_g_, 37.3 °C, was obtained (Table 1, entry 2). From *in situ* IR monitoring, in addition to terpolymer, a notable amount of cyclic propylene carbonate formed. However, traces showed from a kinetic standpoint while the cyclic product formed quickly, once polymerization began there was no further cyclic formation (Fig. S13†). Instead the starting PO monomer continued to insert into the growing polymeric chain. When these ratios were reversed 90 : 10 CHO : PO (Table 1, entry 3), the polymer contained mostly PCHC linkages with a *T*_g_ similar to polycyclohexene carbonate. When CHO was replaced with VCHO in combination with PO (Table 1, entries 1 and 4), a larger proportion of PO was incorporated into the terpolymer. The BPh_3_/PPNCl system did not give cyclic or polymer product when glycidol was used (Table 1, entry 6). Allyl glycidyl ether (AGE) in the presence of CHO or VCHO (Table 1, entries 7 and 9) could be incorporated into terpolymers but only modest amounts of AGE were found in the resulting polymer. All obtained terpolymers were characterized by ^1^H and ^13^C NMR spectroscopy, GPC and DSC (Fig. S9–S26†). DOSY NMR spectroscopy confirmed the incorporation of both epoxides within the same polymeric chain. The terpolymers with alkene functionality (*i.e.* those containing VCHO and AGE) are attractive as they introduce the potential to further modify the polymers.

**Table 1:** Tepolymerizations of epoxides and CO_2_ catalyzed by BPh_3_^a^

Entry	Epoxide A equiv. (%)	Epoxide B equiv. (%)	Monomer incorporation^b^ (A : B)	*M*_n_ c (g mol^−1^)	*Đ* c	*T*_g_ d (°C)
1	CHO (50)	PO (50)	4 : 1	7990	1.03	79.9
2	CHO (10)	PO (90)	1 : 2	7310	1.06	37.3
3	CHO (90)	PO (10)	25 : 1	3760	1.03	110.2
4	VCHO (50)	PO (50)	1.5 : 1	9660	1.08	78.4
5	CHO (50)	Glycidol (50)	No reaction	—	—	—
6	CHO (50)	AGE (50)	1 : 0.1	6080	1.07	72.5
7	CHO (50)	VCHO (50)	1 : 0.5	5120	1.10	109.1
8	VCHO (50)	AGE (50)	2.2 : 1	7540	1.09	76.6

a General reaction conditions unless otherwise indicated: total epoxide (A + B) (0.025 mol), PPNCl (0.124 mmol), BPh_3_ (0.124 mmol), 60 °C, 40 bar CO_2_. All obtained terpolymers contained >99% CO_3_ linkages, no evidence of polyether formation.

b Determined by ^1^H NMR spectroscopy.

c *Đ*, dispersity = *M*_w_/*M*_n_. Determined in THF by GPC equipped with a multiangle light-scattering detector.

d Determined from DSC.

Building upon our initial polycarbonate hydrosilylation results, we then performed ‘one-pot’ hydrosilylation using the CHO/VCHO terpolymer (Table 1, entry 8) following similar procedures to those discussed above (Fig. 2). For the copolymerization step, a catalyst loading of 2.5 mol% BPh_3_ was used, which corresponds to 5 mol% BPh_3_ for the hydrosilylation step (as only 50% VCHO was present). After 24 h the vessel was cooled, depressurized and a mixture of diphenylsilane in dichloromethane was injected into the vessel. The mixture was then heated to 60 °C for 24 h. *Via in situ* IR spectroscopy, we observed a decreased in intensity of the silane bands (2144 and 845 cm^−1^), which plateaued after approx. 12 h and growth of a band at 830 cm^−1^ corresponding to the hydrosilylated product. The hydrosilylated polymer was further characterized by ^1^H, ^13^C, HSQC and refocused INEPT ^29^Si NMR spectroscopy (Fig. S27–S32†). From ^1^H NMR integration of signals for the residual vinyl protons and the aromatic protons (–SiPh_2_), 36% of vinyl groups had been modified. The refocused INEPT ^29^Si NMR spectrum of the product had a resonance at *δ* = −19.32 ppm, *cf. δ* = −33.18 ppm (Ph_2_SiH_2_). From both ^1^H and refocused INEPT ^29^Si NMR it is evident that only one Si–H bond added across the alkene of the terpolymer and hence no cross-linking occurred. From DSC data, a decline in *T*_g_ from 111.6 °C to 47.8 °C was observed for the silylated product (Fig. S33†). From GPC, an increase in molecular weight from 4.31 × 10^3^ g mol^−1^ (*Đ* = 1.07) to 6.20 × 10^3^ g mol^−1^ (*Đ* = 1.12) was observed as well as increased branching in MHS confirmation plots (Fig. S34†).

**Fig. 2:**
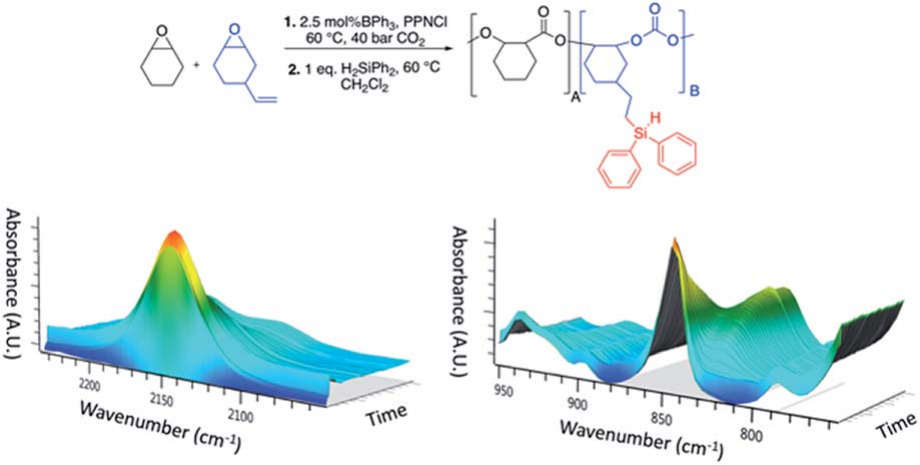
One-pot assisted tandem catalysis to yield silylated-terpolymers. General reaction scheme (top) and three-dimensional plots obtained *via in situ* IR spectroscopy showing a decreased for silane bands and growth of product bands (bottom).

Finally, we set out to evaluate the substrate scope of these transformations by evaluating the reactivity of a hydride terminated polydimethylsiloxane. For the hydride terminated polydimethylsiloxane (DMS-HO3) and PVCHC, the resulting polymer was characterized by ^1^H, ^13^C, refocused INEPT ^29^Si, and H–Si HMQC NMR spectroscopy (Fig. S35–S37†). *Via* integration of the ^1^H NMR spectrum, hydrosilylation has occurred to a similar extent to other hydrosilylations reported herein. We note that only one Si–H group per DMS-HO3 has undergone reaction and no cross-linking between polycarbonate chains was indicated by NMR and DSC data. IR spectra of the hydrosilylated-polycarbonate showed new bands at *ν* = 1013, 907 and 788 cm^−1^ corresponding to O–Si–O, Si–H and Si–CH_3_ groups respectively (Fig. S38†). From GPC, there was a moderate increase in molecular weight and no significant change in *Đ*. There was a decline in the slope of the MHS plot indicating a higher degree of branching in the final product (Fig. S39†). DSC analysis demonstrated a slight increase in *T*_g_ from 99.0 °C to 104.6 °C. The residual unreacted Si–H bonds in the functionalized polymer introduces further functionality potential. For example, BCF has been reported to catalyze the addition of Si–H bonds onto silica derived materials.^39^

In summary, we report the first example of BPh_3_ catalyzed hydrosilylation of perfectly alternating PVCHC in a tandem catalytic manner. These reactions were monitored by *in situ* IR spectroscopy, which demonstrated the addition of the Si–H bond across the pendent alkenes in the polymer. In an attempt to build new classes of polymeric materials, we showed the ability of BPh_3_ to catalyze the terpolymerization of CO_2_ and several epoxide combinations, yielding products with *T*_g_ values from 37.3 °C to 110.2 °C, which we could then functionalize in a similar one-pot manner as above. Finally, we evaluated the reactivity of a polymeric hydride terminated siloxane which can serve as a precursor for silica surface modification. Using the results in-hand, we will work towards developing sustainable surface functionalized materials in the future.

## Conflicts of interest

There are no conflicts to declare.

## Supplementary Material

RA-009-C9RA05947D-s001
