# Supplementary material for: Functionalized polycarbonates via triphenylborane catalyzed polymerization-hydrosilylation
Source: RSC Adv. 2019 Aug 23;9(46):26542–6. doi: 10.1039/c9ra05947d (PMC9070434; doi:10.1039/c9ra05947d)
Supplement: RA-009-C9RA05947D-s001 [file RA-009-C9RA05947D-s001.pdf]

This journal is © The Royal Society of Chemistry 2019

Functionalized Polycarbonates via Triphenylborane Catalyzed Polymerization-Hydrosilylation

Kori A. Andrea and Francesca M. Kerton*

Department of Chemistry, Memorial University of Newfoundland, St. John's, NL, A1B 3X7, Canada

fkerton@mun.ca

Table of Contents

1. **General experimental conditions**
2. **One-pot formation of silyl-modified polycarbonate**
3. **General procedure for terpolymerizations**
4. **Polydimethylsiloxane-functionalized polyvinylcyclohexene carbonate**
5. **Figure S1** ^{1}H NMR (300 MHz, $CDCl_3$, 298 K) spectrum of isolated poly(vinylcyclohexenecarbonate) [PVCHC].
6. **Figure S2** ^{1}H NMR (300 MHz, $CDCl_3$, 298 K) spectrum of isolated PVCHC (top) and isolated silylated PVCHC (bottom). 10% of vinyl groups have been silylated (see Fig. S6 for expanded view).
7. **Figure S3** $^{13}C\{^{1}H\}$ NMR (125 MHz, $CDCl_3$, 298 K) spectrum of isolated PVCHC (top) and isolated silylated PVCHC (bottom).
8. **Figure S4** HSQC 2-D NMR spectrum of isolated silylated PVCHC. x-axis shows ^{1}H NMR spectrum (300 MHz, $CDCl_3$, 298 K) and y-axis shows $^{13}C\{^{1}H\}$ NMR spectrum (75 MHz, $CDCl_3$, 298 K) of isolated PVCHO.
9. **Figure S5** HMQC 2-D NMR spectrum of isolated silylated PVCHC. x-axis shows ^{1}H NMR spectrum (300 MHz, $CDCl_3$, 298 K) and y-axis shows ^{29}Si NMR spectrum (60 MHz, $CDCl_3$, 298 K) of the isolated polymer. J(H-Si)= 10 Hz
10. **Figure S6** Integrated ^{1}H NMR spectrum of silylated PVCHC. Normalized 1 SiMe_2 group, 6H, which corresponds 8.78 C$H_{(B)}$ residual vinyl groups. % silyl = m/(m+n) *100% = 1/(1+8.78) *100% = 10.2% functionalization.
11. **Figure S7** GPC chromatogram showing light scattering trace of isolated PVCHC (at 5 mol% BPh_3) and silylated PVCHC from one-pot synthesis.
12. **Figure S8** Mark-Houwink-Sakurada plot for isolated PVCHC (at 5 mol% BPh_3) and silylated PVCHC from one-pot synthesis.
13. **Figure S9** Integrated ^{1}H NMR (300 MHz, $CDCl_3$, 298 K) spectrum of PO/CHO/CO_2 terpolymer from Table 1, entry 1.
14. **Figure S10** $^{13}C\{^{1}H\}$ NMR (125 MHz, $CDCl_3$, 298 K) spectrum of PO/CHO/CO_2 terpolymer from Table 1, entry 1.
15. **Figure S11** Zoom $^{13}C\{^{1}H\}$ NMR (125 MHz, $CDCl_3$, 298 K) spectrum of PO/CHO/CO_2 terpolymer from Table 1, entry 1, showing isotacticity of PCHC region.
16. **Figure S12** Zoom $^{13}C\{^{1}H\}$ NMR (125 MHz, $CDCl_3$, 298 K) spectrum of PO/CHO/CO_2 terpolymer from Table 1, entry 1, showing isotacticity of PPC region.

17. **Figure S13** 3D React IR plot from Table 1, entry 1 showing major product is polycarbonate while cyclic carbonate as the minor product.

18. **Figure S14** Integrated ^{1}H NMR (300 MHz, $CDCl_3$, 298 K) spectrum of PO/CHO/CO_2 terpolymer from Table 1, entry 2.

19. **Figure S15** $^{13}C\{^{1}H\}$ NMR (125 MHz, $CDCl_3$, 298 K) spectrum of PO/CHO/CO_2 terpolymer from Table 1, entry 2.

20. **Figure S16** Integrated ^{1}H NMR (300 MHz, $CDCl_3$, 298 K) spectrum of PO/CHO/CO_2 terpolymer from Table 1, entry 3.

21. **Figure S17** $^{13}C\{^{1}H\}$ NMR (125 MHz, $CDCl_3$, 298 K) spectrum of PO/CHO/CO_2 terpolymer from Table 1, entry 3.

22. **Figure S18** Integrated ^{1}H NMR (300 MHz, $CDCl_3$, 298 K) spectrum of PO/VCHO/CO_2 terpolymer from Table 1, entry 4.

23. **Figure S19** $^{13}C\{^{1}H\}$ NMR (125 MHz, $CDCl_3$, 298 K) spectrum of PO/VCHO/CO_2 terpolymer from Table 1, entry 4.

24. **Figure S20** Integrated ^{1}H NMR (300 MHz, $CDCl_3$, 298 K) spectrum of CHO/AGE/CO_2 terpolymer from Table 1, entry 5.

25. **Figure S21** $^{13}C\{^{1}H\}$ NMR (125 MHz, $CDCl_3$, 298 K) spectrum of CHO/AGE/CO_2 terpolymer from Table 1, entry 5.

26. **Figure S22** Integrated ^{1}H NMR (300 MHz, $CDCl_3$, 298 K) spectrum of CHO/VCHO/CO_2 terpolymer from Table 1, entry 6.

27. **Figure S23** $^{13}C\{^{1}H\}$ NMR (125 MHz, $CDCl_3$, 298 K) spectrum of CHO/VCHO/CO_2 terpolymer from Table 1, entry 6.

28. **Figure S24** Integrated ^{1}H NMR (300 MHz, $CDCl_3$, 298 K) spectrum of VCHO/AGE/CO_2 terpolymer from Table 1, entry 7.

29. **Figure S25** $^{13}C\{^{1}H\}$ NMR (125 MHz, $CDCl_3$, 298 K) spectrum of VCHO/AGE/CO_2 terpolymer from Table 1, entry 7.

30. **Figure S26** Representative DOSY NMR spectra of obtained terpolymer (CO_2/CHO/AGE) from Table 1, entry 9. (^{1}H NMR 500 MHz, $CDCl_3$, 298 K)

31. **Figure S27** ^{1}H NMR (300 MHz, $CDCl_3$, 298 K) spectrum of pure Ph_2SiH_2 (top) and resulting functionalized terpolymer (CHO/VCHO/CO_2) (bottom). 36.4% of vinyl groups have been silylated (see Fig. S31 for expanded view).

32. **Figure S28** $^{13}C\{^{1}H\}$ NMR (125 MHz, $CDCl_3$, 298 K) spectrum of pure Ph_2SiH_2 (top) and resulting functionalized terpolymer (CHO/VCHO/CO_2) (bottom).

33. **Figure S29** Refocused INEPT ^{29}Si NMR (60 MHz, $CDCl_3$, 298 K) spectrum of isolated PVCHC (top) and isolated silylated terpolymer (CHO/VCHO/CO_2) (bottom).

34. **Figure S30** HSQC 2-D NMR spectrum of isolated silylated terpolmer (CHO/VCHO/CO_2). x-axis shows ^{1}H NMR spectrum (300 MHz, $CDCl_3$, 298 K) and y-axis shows $^{13}C\{^{1}H\}$ NMR spectrum (75 MHz, $CDCl_3$, 298 K) of the isolated polymer.

35. **Figure S31** Integrated ^{1}H NMR (300 MHz, $(CD_3)_2CO$, 298 K) spectrum of silylated PVCHC. Normalized 1 SiAr_2 group, 10H, which corresponds 1.75 $CH_{(B)}$ residual vinyl groups. % silyl = m/(m+n) *100% = 1/(1+1.75) *100% = 36.4% functionalization.

36. **Figure S32** HMQC 2-D NMR spectrum of isolated silylated terpolmer ($CHO/VCHO/CO_2$). x-axis shows ^{1}H NMR spectrum (300 MHz, $CDCl_3$, 298 K) and y-axis shows ^{29}Si NMR spectrum (60 MHz, $CDCl_3$, 298 K) of the isolated polymer. J(H-Si)= 300 Hz
37. **Figure S33** DSC of native terpolymer (CO_2/CHO/VCHO) from one pot reaction (blue) and after silane functionalization (red). Glass transition temperatures taken from the 3rd cycle.
38. **Figure S34** Mark-Houwink-Sakurada plot for isolated terpolymer ($CHO/VCHO/CO_2$) and the corresponding silylated terpolymer.
39. **Figure S35** ^{1}H NMR (300 MHz, $CDCl_3$, 298 K) spectrum of pure DMS-HO3 (top) and resulting functionalized PVCHC (bottom).
40. **Figure S36** Refocused INEPT ^{29}Si NMR (60 MHz, $CDCl_3$, 298 K) spectrum of pure DMS-HO3 (top) and resulting functionalized PVCHC (bottom).
41. **Figure S37** HMQC 2-D NMR spectrum of isolated DMS-HO3 functionalized PVCHC x-axis shows ^{1}H NMR spectrum (300 MHz, $CDCl_3$, 298 K) and y-axis shows ^{29}Si NMR spectrum (60 MHz, $CDCl_3$, 298 K) of the isolated polymer. J(H-Si)= 10 Hz
42. **Figure S38** Stacked FTIR spectra of isolated PVCHC (red), DMS-HO3 (blue) and DMS-HO3 functionalized PVCHC (black)
43. **Figure S39** Mark-Houwink-Sakurada plot for isolated PVCHC (green) and DMS-HO3 functionalized PVCHC (red)

General experimental conditions. Unless otherwise stated, all experimental procedures were performed using an MBraun Labmaster glove box or under dry oxygen-free nitrogen using Schlenk techniques. Propylene oxide and bis(triphenyphosphine)iminium chloride (PPNCl) were purchased from Alfa Aesar. Cyclohexene oxide, vinyl cyclohexene oxide, allyl glycidol ether, epichlorohydrin and glycidol were purchased from Sigma Aldrich. All epoxides were dried over CaH_2 and distilled under vacuum. Phenyldimethylsilane, diphenylsilane and hydride terminated polydimethylsiloxane (DMS-H03) were purchased from Gelest and used without further purification. All solvents were dried and degassed using an MBraun Manual Solvent Purification system. Triphenylborane (BPh_3) was purchased from Strem Chemicals and used without further purification. Caution should be taken when operating high pressure equipment.

Instrumentation. ^1H and $^{13}C\{^1H\}$, ^{29}Si NMR spectra were recorded on a Bruker Avance 300 MHz spectrometer at 25 °C (frequencies were ^{13}C, 75.43 MHz; ^{29}Si, 59.60 MHz). All NMR spectra were obtained in $CDCl_3$ or $(CD_3)_2CO$ purchased from Cambridge Isotope Laboratories, Inc. ^1H and ^{13}C NMR spectra were referenced using the residual proton and ^{13}C resonances of the solvent. Refocused INEPT ^{29}Si was referenced to external tetramethylsilane (TMS, δ = 0 ppm). All cyclization and copolymerization reactions were carried out in a 100 mL stainless steel reactor vessel (Parr Instrument Company) equipped with a silicon sensor (SiComp), mechanical stirrer and a heating mantel. For kinetic measurements, the Si sensor was connected to a ReactIR 15 base unit (Mettler-Toledo) through a DS silver-halide Fiber-to-Sentinel conduit. The vessel was baked at 100 °C under vacuum overnight prior to any experiment. Gel permeation chromatography (GPC) analysis was performed on a set-up consisting of a miniDawn TREOS light scattering detector, a Viscostar-II viscometer, and an Optilab T-rEX differential refractive index detector (Wyatt Technology) connected to an Agilent Infinity 1260 HPLC system equipped with two Phenogel 10^3 Å 300 × 4.60 mm columns with THF as eluent. Samples were prepared in THF at a concentration of 4 mg mL^{-1}, filtered through a 0.2 µm syringe filter, and analyzed at a flow rate of 0.3 mL min^{-1} at 25 °C. The values of dn/dc were calculated online (columns detached) assuming 100% mass recovery using the Astra 6 software package (Wyatt Technologies). Glass transition temperatures (T_g) were obtained on a Mettler Toledo DSC $Star^e$ system equipped with a Julabo FT 100 immersion cooling system for low temperatures (-100 °C +20 °C). Samples were weighed into 40 μL aluminum pans and exposed to 3 heating cycles from 0 to 200 °C at a rate of 10 °C min^{-1}, with a hold time of 2 min at both 0 °C and 200 °C in each cycle. The reported T_g values were determined using data from the third heating cycle.

One-pot formation of silyl-modified polycarbonate. Stock solutions of BPh_3 (24.4 mg mL^{-1}) and PPNCl in toluene were combined and solvent was removed under vacuum. The solids were dissolved in 3 mL of dichloromethane and the solution was injected into the pressure vessel. The dichloromethane removed under vacuum, before the desired amount of the vinylcyclohexene oxide monomer was injected into the pressure vessel. The vessel was pressurized with CO_2, heated to the desired temperature and mechanically stirred for 24 h. After 24 h, the vessel was cooled to room temperature and slowly depressurized into a fumehood. A solution of phenyldimethylsilane (2.74 g, 20.1 mmol) in 20 mL of dichloromethane was injected into the vessel. The reaction mixture was heated to 40 °C for 4 days. However, after observing no change in signal intensity for phenyldimethylsilane, the temperature was increased to 60 °C for 24 h and IR bands corresponding to the silylated polymer grew in intensity (Figure 1). After 24 h, the vessel was cooled, the solution was taken out of the pressure vessel, concentrated and the polymer precipitated using cold, acidified methanol.

General procedure for terpolymerizations. Stock solutions of BPh_3 (24.4 mg mL^{-1}) and PPNCl in toluene were combined and the solvent was removed under vacuum. The solids were dissolved in 3 mL of dichloromethane and the solution was injected into the pressure vessel. The dichloromethane removed under vacuum, before the desired epoxide mixture was injected into the vessel. The vessel was pressurized with CO_2, heated to the desired temperature and mechanically stirred for 24 h. After 24 h, the vessel was cooled to room temperature and slowly depressurized into a fumehood. The crude product was dissolved in minimal dichloromethane and precipitated in cold acidified methanol.

Polydimethylsiloxane-functionalized polyvinylcyclohexene carbonate. In a glovebox, purified PVCHC (0.20 g, 1.01 mmol vinyl units) and BPh_3 (0.012 g, 0.050 mmol) was dissolved in 10 mL of dichloromethane. To this, hydride terminated polydimethylsiloxane (2.52 g, 5.04 mmol) was added dropwise with stirring. The vial was sealed and allowed to stir overnight at room temperature. The crude produced was exposed to air, and precipitated in cold acidified methanol.

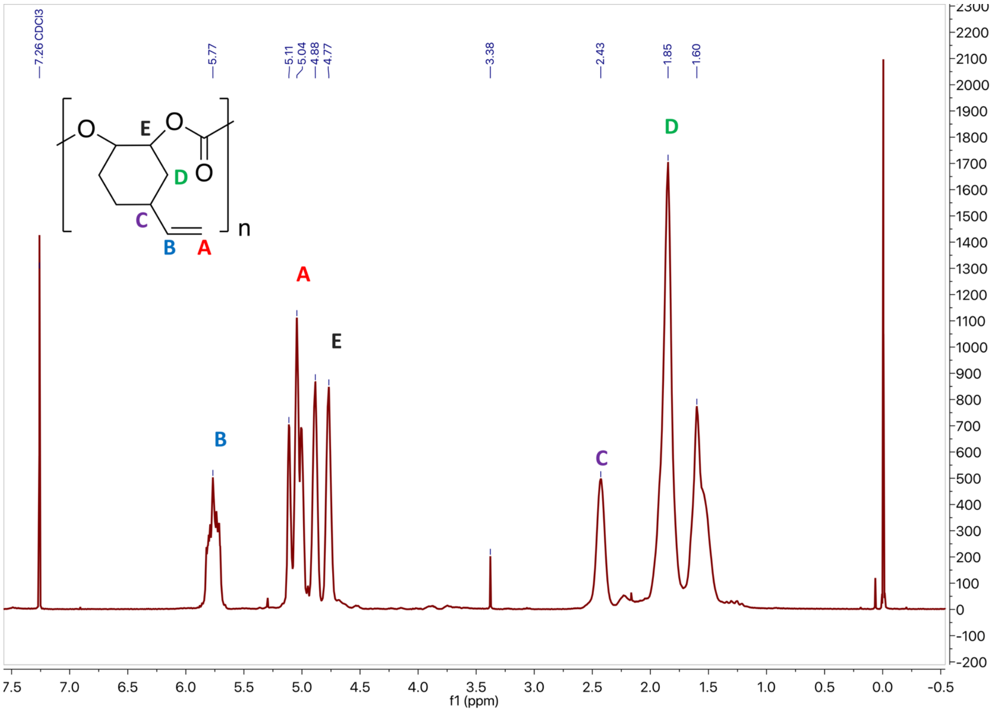

Figure S1. ^{1}H NMR (300 MHz, $CDCl_3$ 298 K) spectrum of isolated poly(vinylcyclohexenecarbonate) [PVCHC].

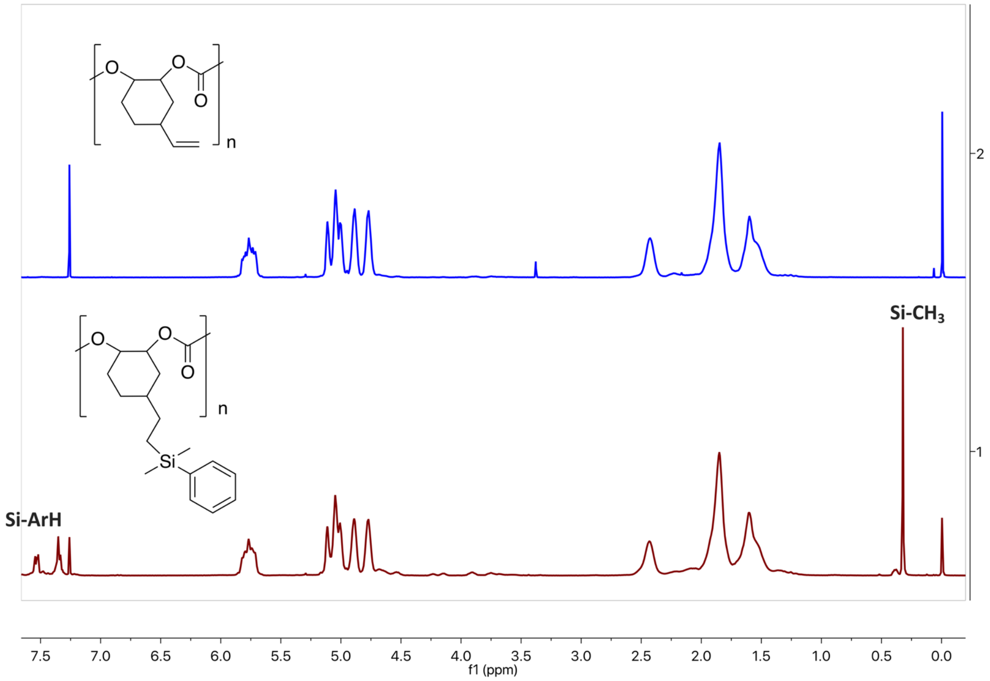

Figure S2. ^{1}H NMR (300 MHz, $CDCl_3$, 298 K) spectrum of isolated PVCHC (top) and isolated silylated PVCHC (bottom). 10% of vinyl groups have been silylated (see Fig. S6 for expanded view).

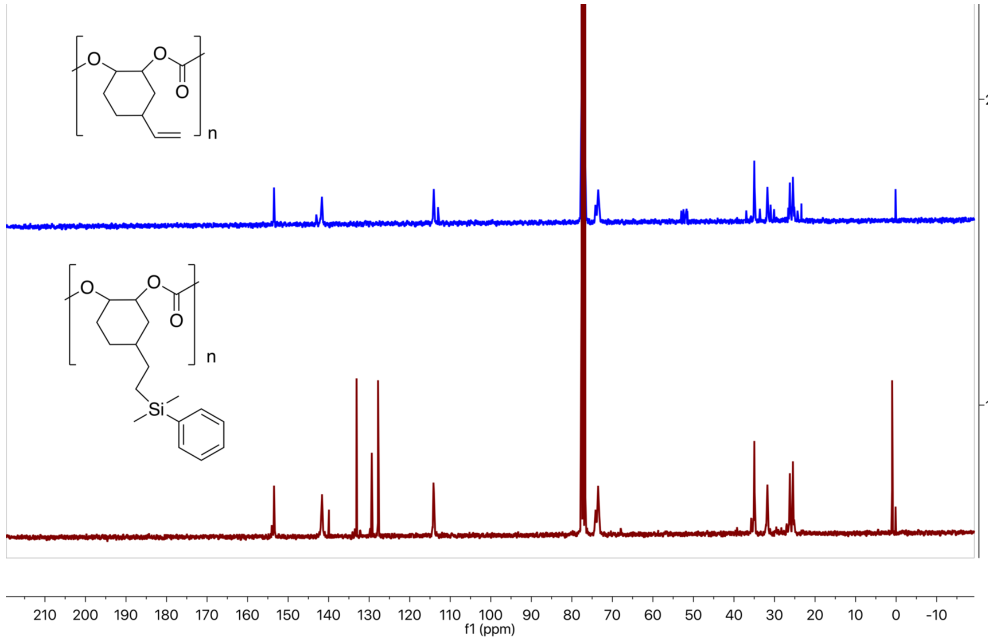

Figure S3. ^{13}C{^{1}H} NMR (125 MHz, $CDCl_{31}$, 298 K) spectrum of isolated PVCHC (top) and isolated silylated PVCHC (bottom).

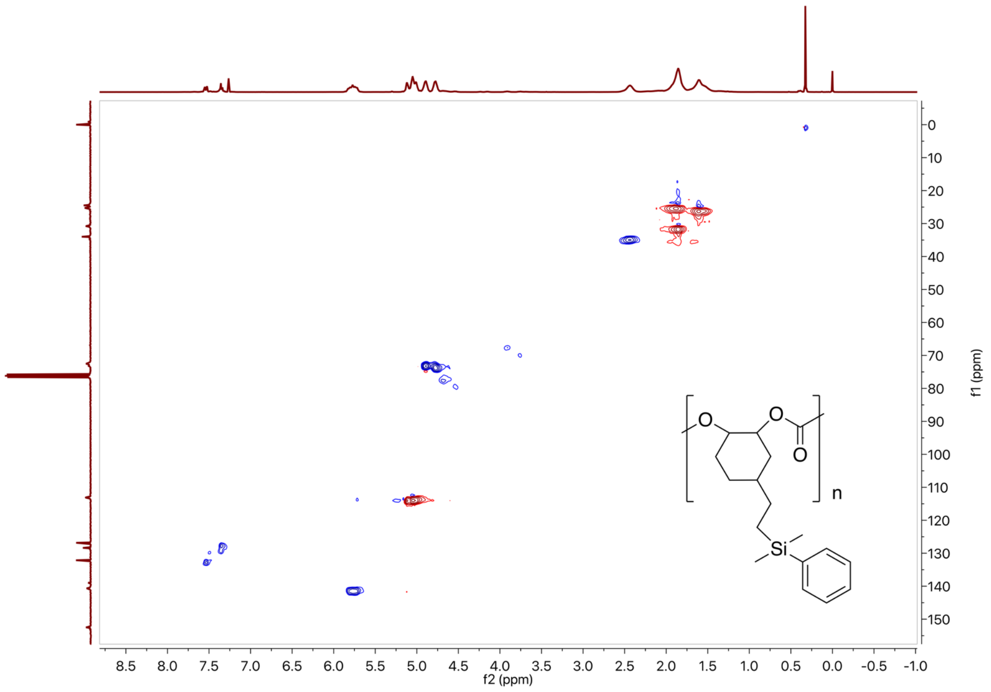

Figure S4. HSQC 2-D NMR spectrum of isolated silylated PVCHC. x-axis shows ^{1}H NMR spectrum (300 MHz, $CDCl_3$, 298 K) and y-axis shows $^{13}C\{^{1}H\}$ NMR spectrum (75 MHz, $CDCl_3$, 298 K) of isolated PVCHC.

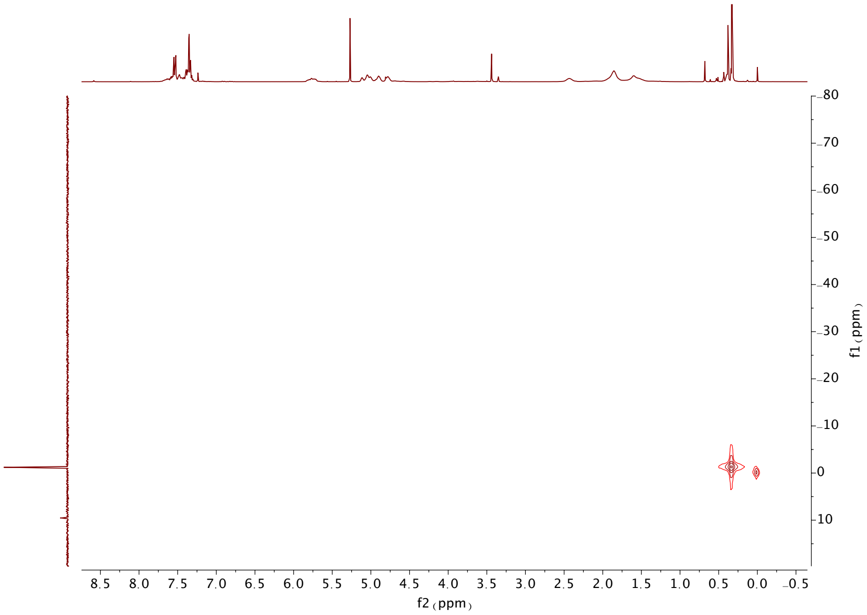

Figure S5. HMQC 2-D NMR spectrum of isolated silylated PVCHC. x-axis shows ^{1}H NMR spectrum (300 MHz, $CDCl_3$, 298 K) and y-axis shows ^{29}Si NMR spectrum (60 MHz, $CDCl_3$, 298 K) of the isolated polymer. J(H-Si)=10 Hz. Cross-peak at 0.0 ppm is TMS.

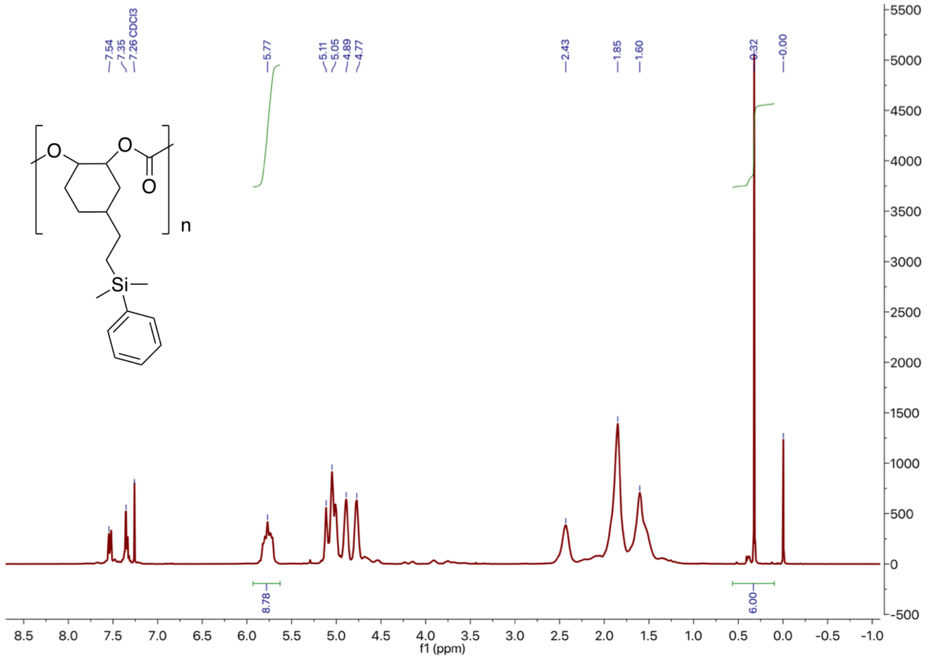

Figure S6. Integrated ^{1}H NMR spectrum of silylated PVCHC. Normalized 1 Si*Me*$_2$ group, 6H, which corresponds 8.78 C*H*$_{(B)}$ residual vinyl groups. % silyl = m/(m+n) *100% = 1/(1+8.78) *100% = 10.2% functionalization.

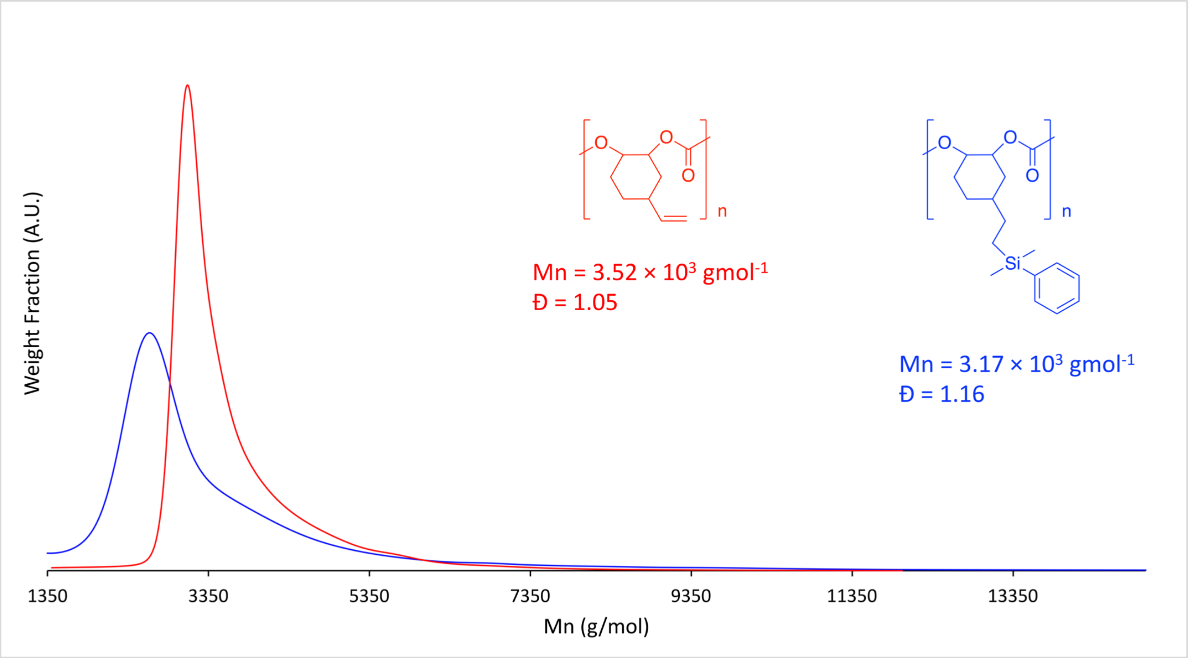

Figure S7. GPC chromatogram showing light scattering trace of isolated PVCHC (at 5 mol% BPh_3) and silylated PVCHC from one-pot synthesis.

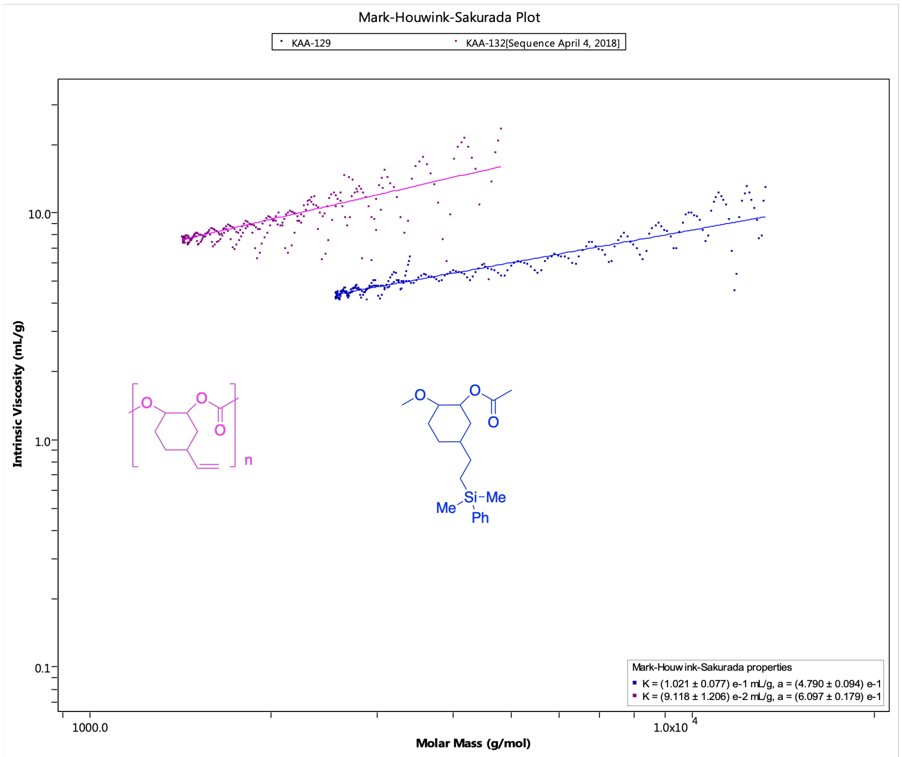

Figure S8. Mark-Houwink-Sakurada plot for isolated PVCHC (at 5 mol% BPh_3) and silylated PVCHC from one-pot synthesis.

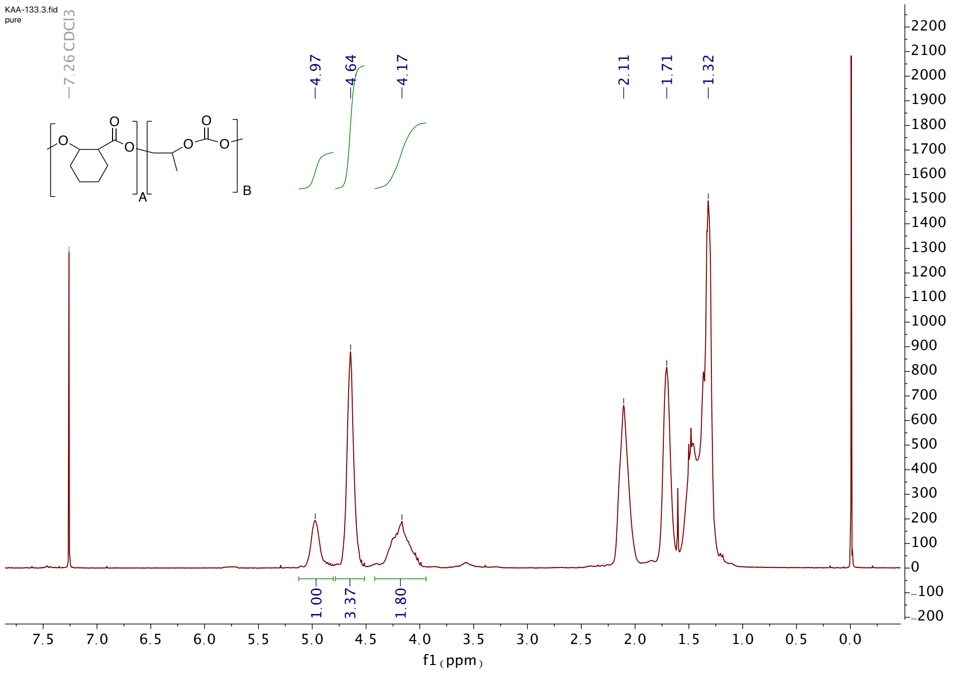

Figure S9. Integrated ^{1}H NMR (300 MHz, $CDCl_3$, 298 K) spectrum of PO/CHO/CO_2 terpolymer from Table 1, entry 1.

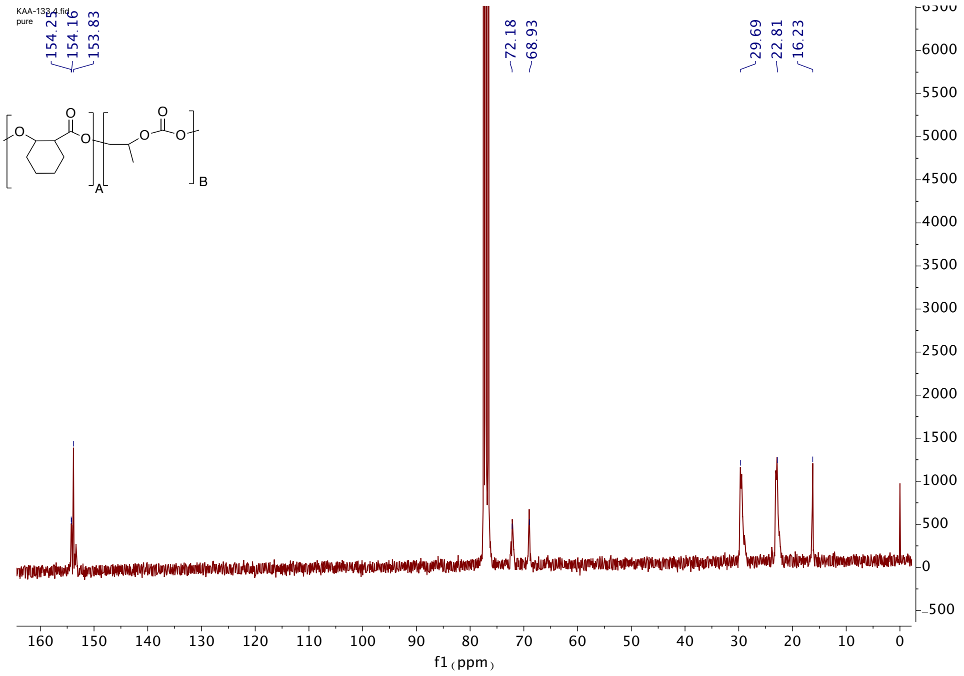

Figure S10. $^{13}C\{^{1}H\}$ NMR (125 MHz, $CDCl_3$, 298 K) spectrum of PO/CHO/CO_2 terpolymer from Table 1, entry 1.

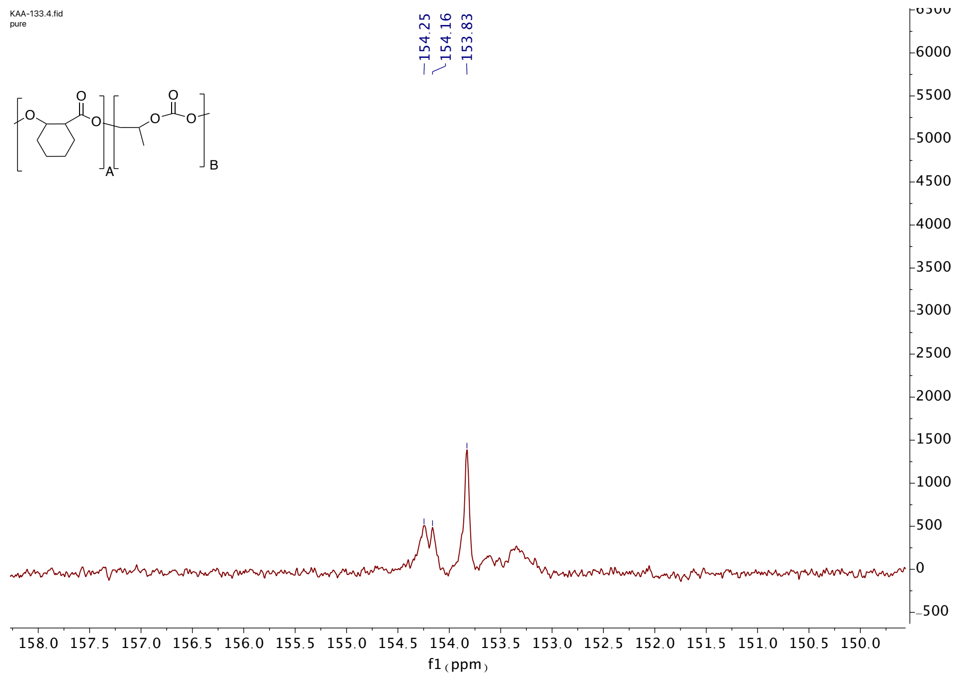

Figure S11. Zoom $^{13}C\{^1H\}$ NMR (125 MHz, $CDCl_3$, 298 K) spectrum of PO/CHO/CO_2 terpolymer from Table 1, entry 1, showing tacticity of PCHC region.

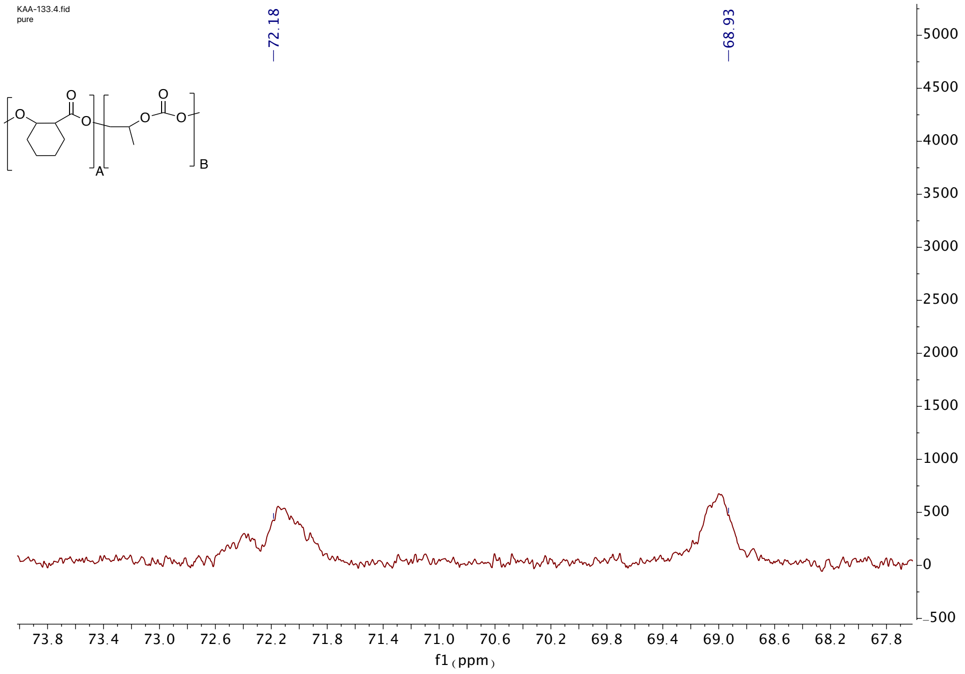

Figure S12. Zoom $^{13}C\{^1H\}$ NMR (125 MHz, $CDCl_3$, 298 K) spectrum of PO/CHO/CO_2 terpolymer from Table 1, entry 1, showing tacticity of PPC region.

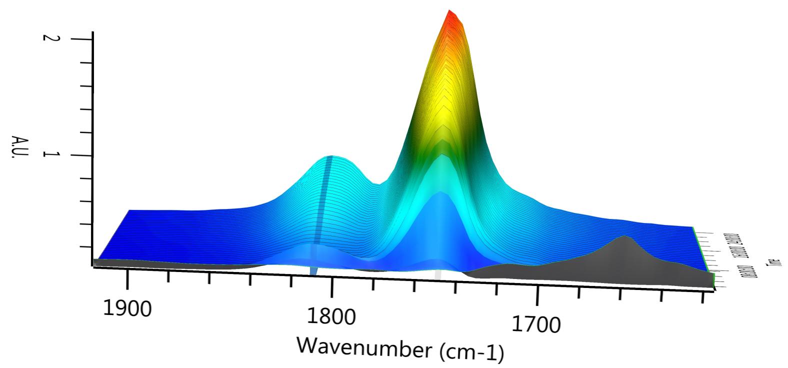

Figure S13. 3D React IR plot from Table 1, entry 1 showing major product is polycarbonate (ν = 1750 cm^{-1}) while cyclic carbonate (ν = 1809 cm^{-1}) is the minor product.

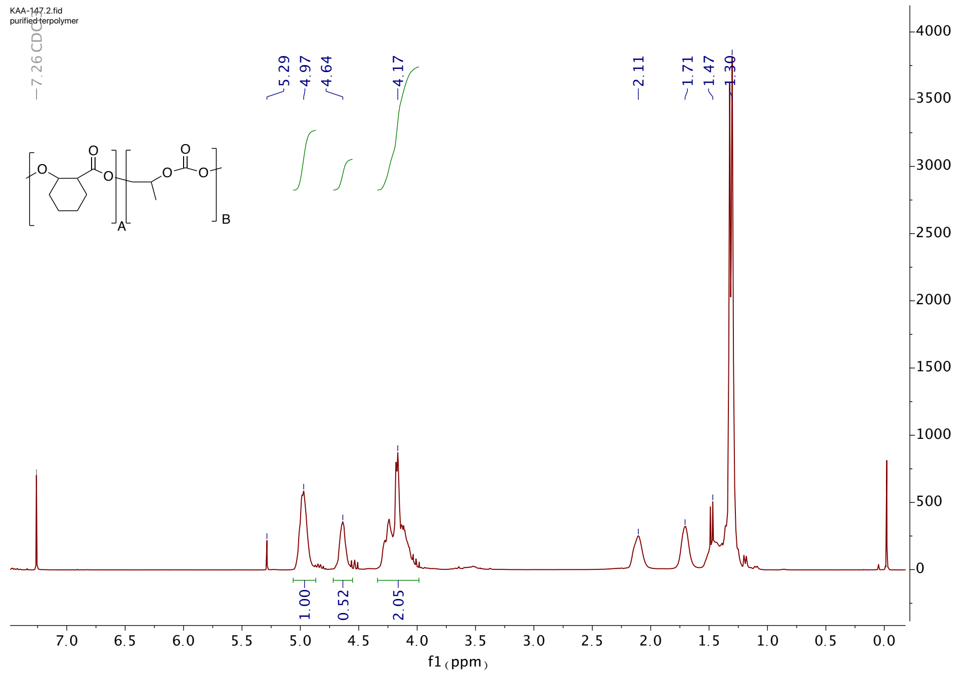

Figure S14. Integrated ^{1}H NMR (300 MHz, $CDCl_3$, 298 K) spectrum of PO/CHO/CO_2 terpolymer from Table 1, entry 2.

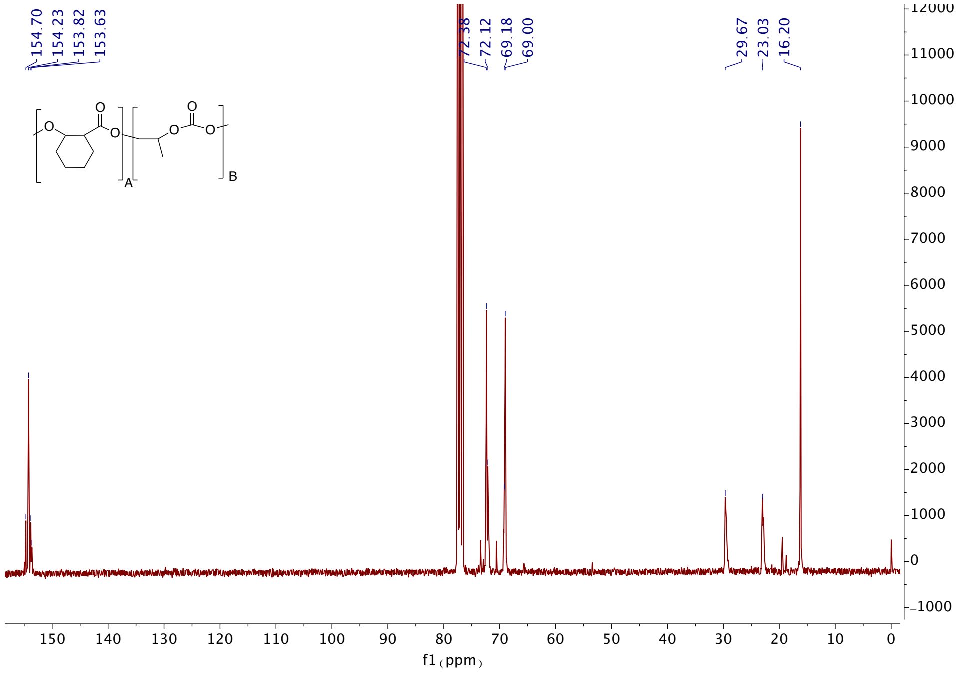

Figure S15. $^{13}C\{^1H\}$ NMR (125 MHz, $CDCl_3$-*d₁*, 298 K) spectrum of PO/CHO/CO_2 terpolymer from Table 1, entry 2.

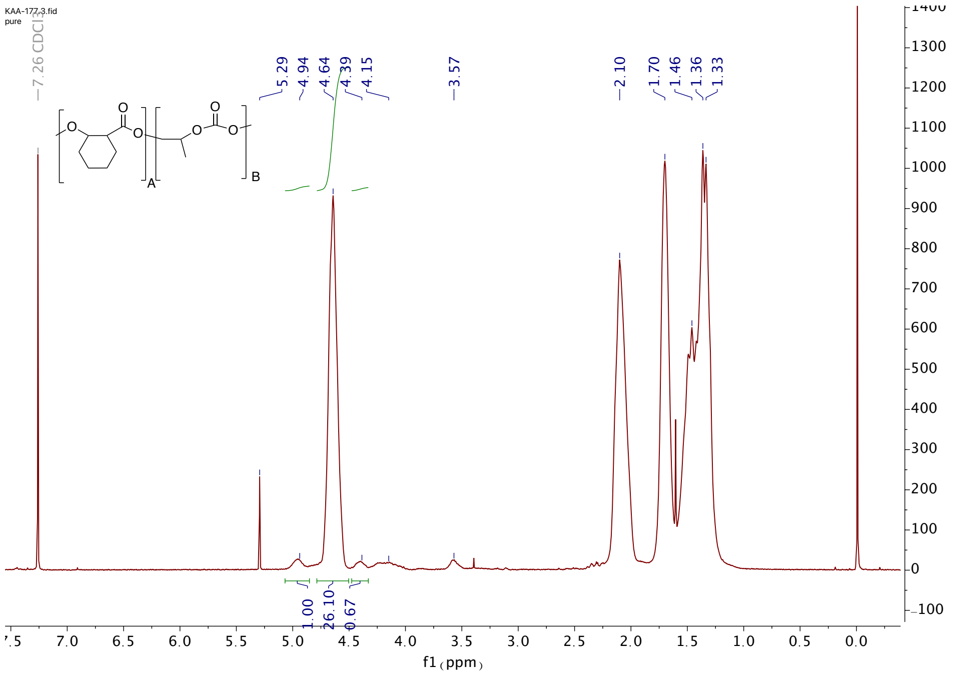

Figure S16. Integrated ^1H NMR (300 MHz, $CDCl_3$, 298 K) spectrum of PO/CHO/CO_2 terpolymer from Table 1, entry 3.

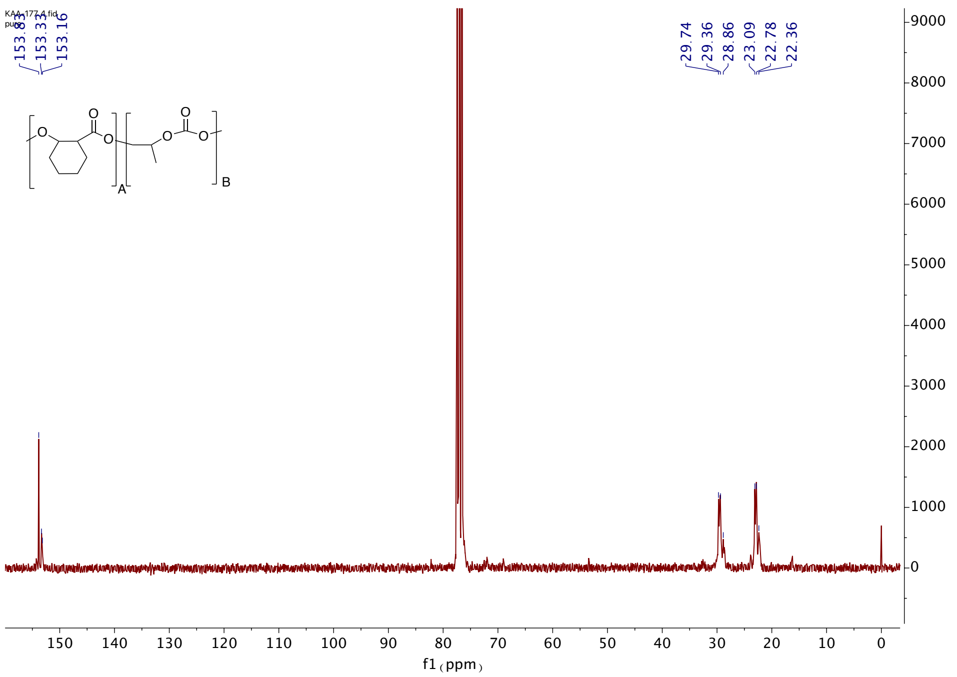

Figure S17. $^{13}C\{^{1}H\}$ NMR (125 MHz, $CDCl_3$, 298 K) spectrum of PO/CHO/CO_2 terpolymer from Table 1, entry 3.

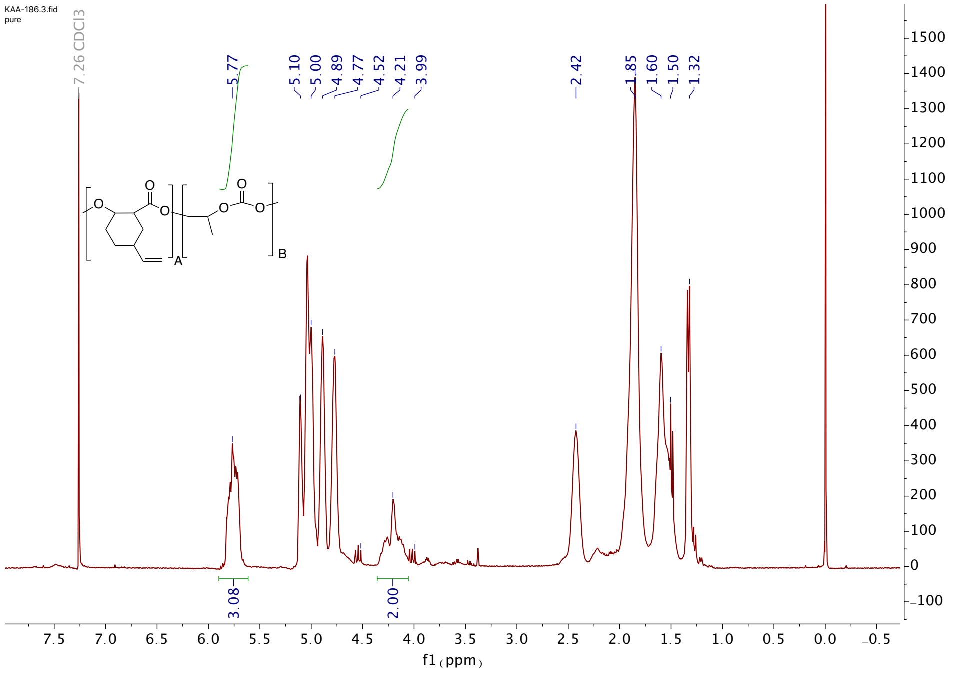

Figure S18. Integrated ^{1}H NMR (300 MHz, $CDCl_3$, 298 K) spectrum of PO/VCHO/CO_2 terpolymer from Table 1, entry 4.

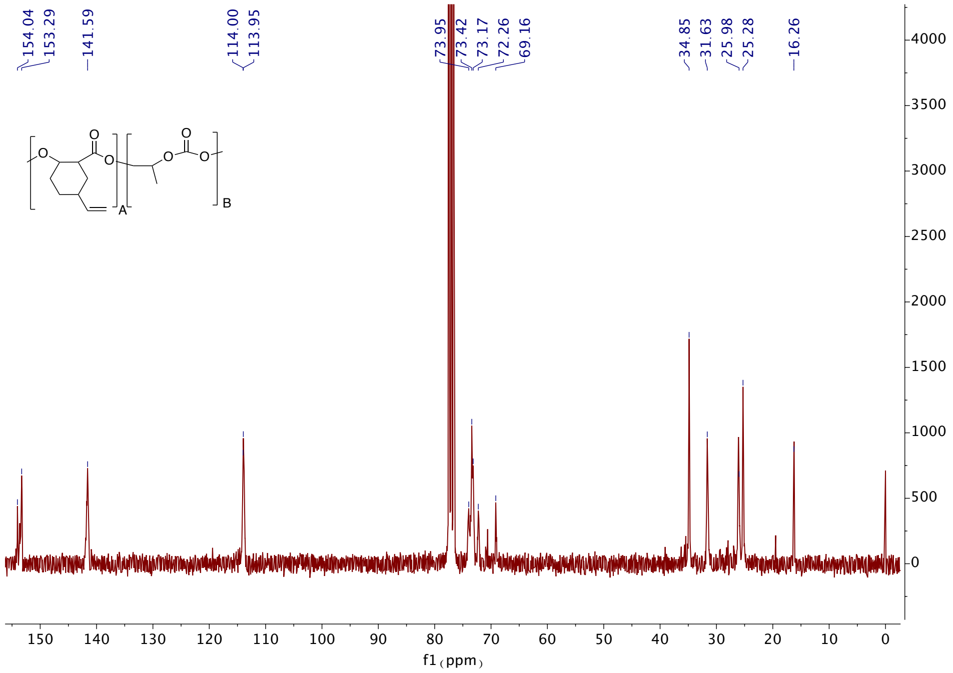

Figure S19. $^{13}C\{^{1}H\}$ NMR (125 MHz, $CDCl_3$, 298 K) spectrum of PO/VCHO/CO_2 terpolymer from Table 1, entry 4.

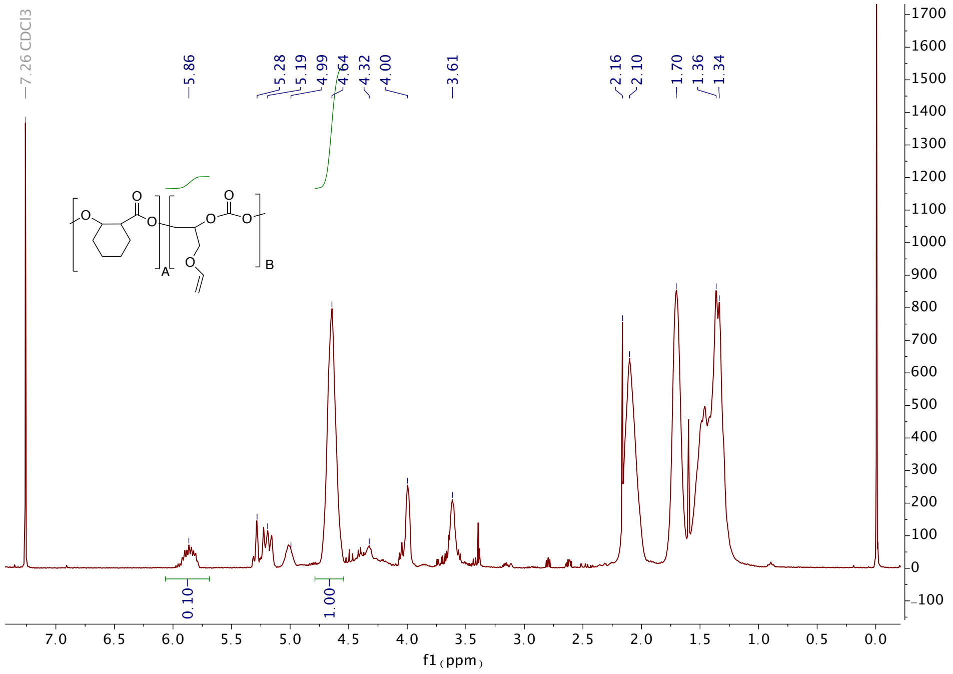

Figure S20. Integrated ^{1}H NMR (300 MHz, $CDCl_3$, 298 K) spectrum of CHO/AGE/CO_2 terpolymer from Table 1, entry 5.

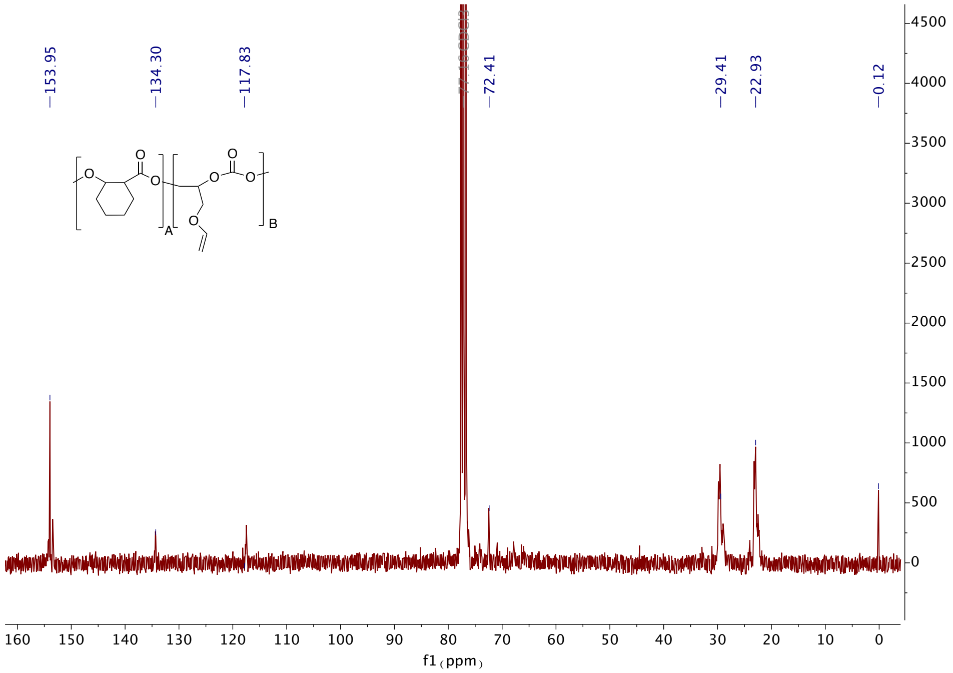

Figure S21. $^{13}C\{^1H\}$ NMR (125 MHz, $CDCl_3$, 298 K) spectrum of CHO/AGE/CO_2 terpolymer from Table 1, entry 5.

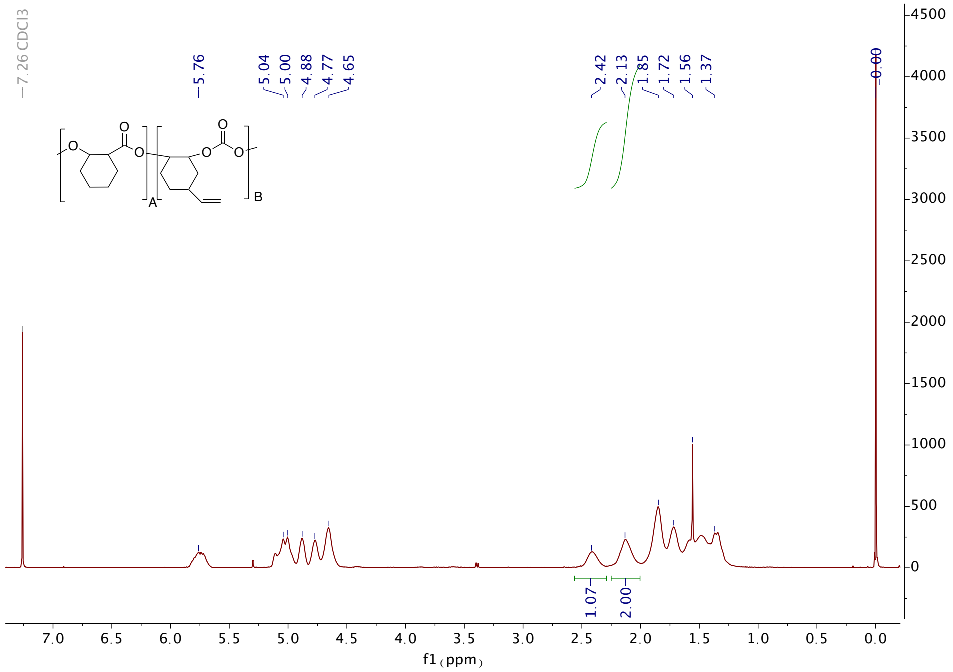

Figure S22. Integrated ^1H NMR (300 MHz, $CDCl_3$, 298 K) spectrum of CHO/VCHO/CO_2 terpolymer from Table 1, entry 6.

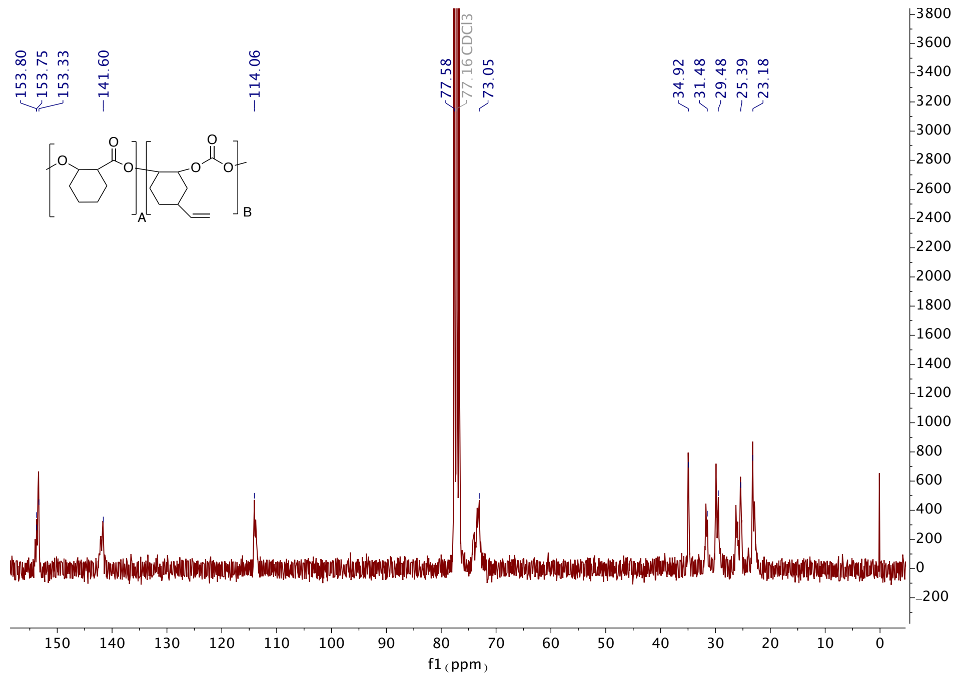

Figure S23. $^{13}C\{^1H\}$ NMR (125 MHz, $CDCl_3$, 298 K) spectrum of CHO/VCHO/CO_2 terpolymer from Table 1, entry 6.

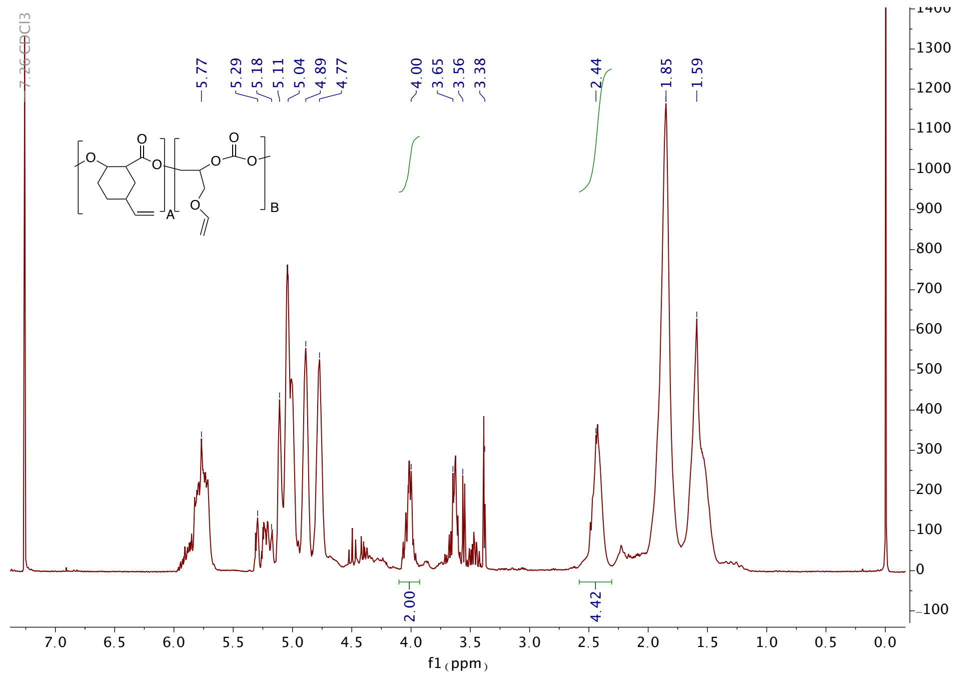

Figure S24. Integrated ^1H NMR (300 MHz, $CDCl_3$, 298 K) spectrum of VCHO/AGE/CO_2 terpolymer from Table 1, entry 7.

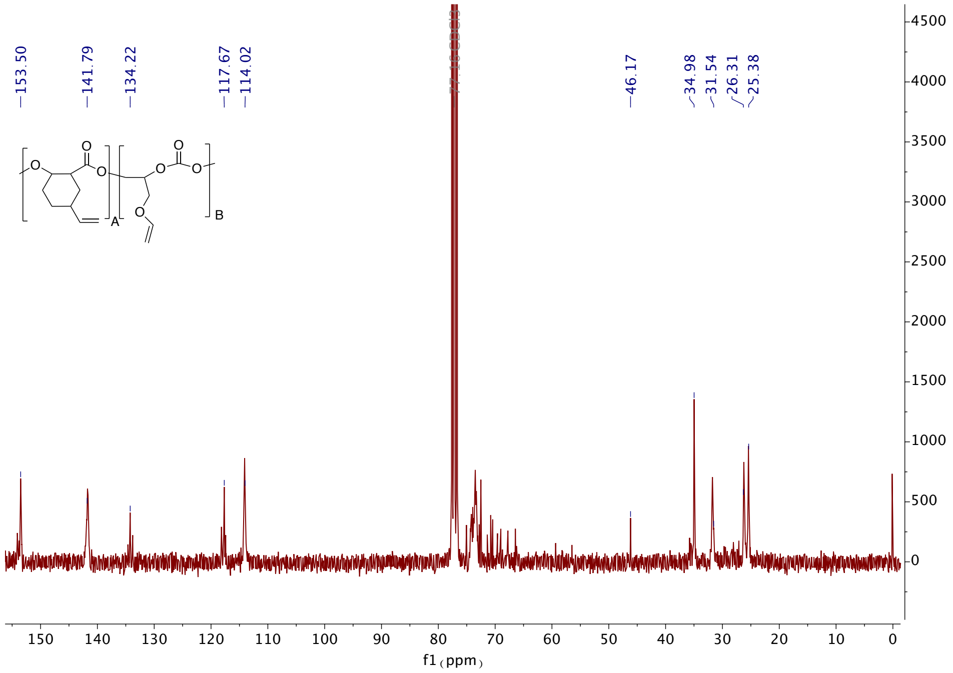

Figure S25. $^{13}C\{^1H\}$ NMR (125 MHz, $CDCl_3$, 298 K) spectrum of VCHO/AGE/CO_2 terpolymer from Table 1, entry 7.

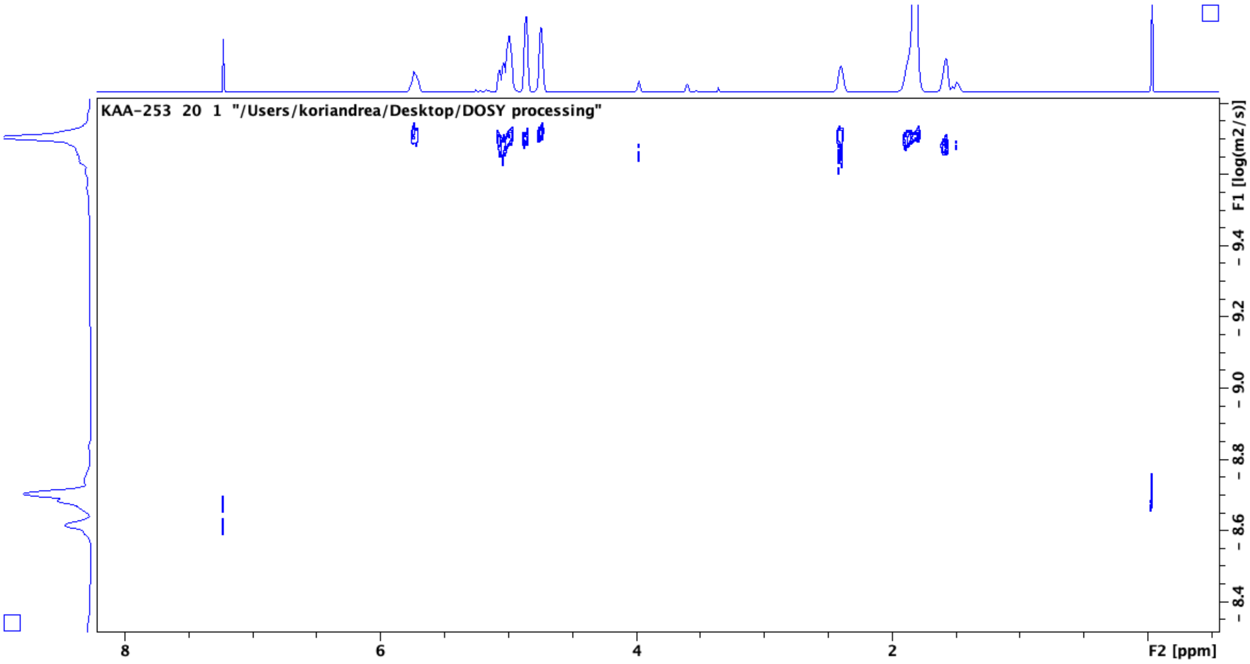

Figure S26. Representative DOSY NMR spectra of obtained terpolymer (CO_2/CHO/AGE) from Table 1, entry 9. (^1H NMR 500 MHz, $CDCl_3$, 298 K)

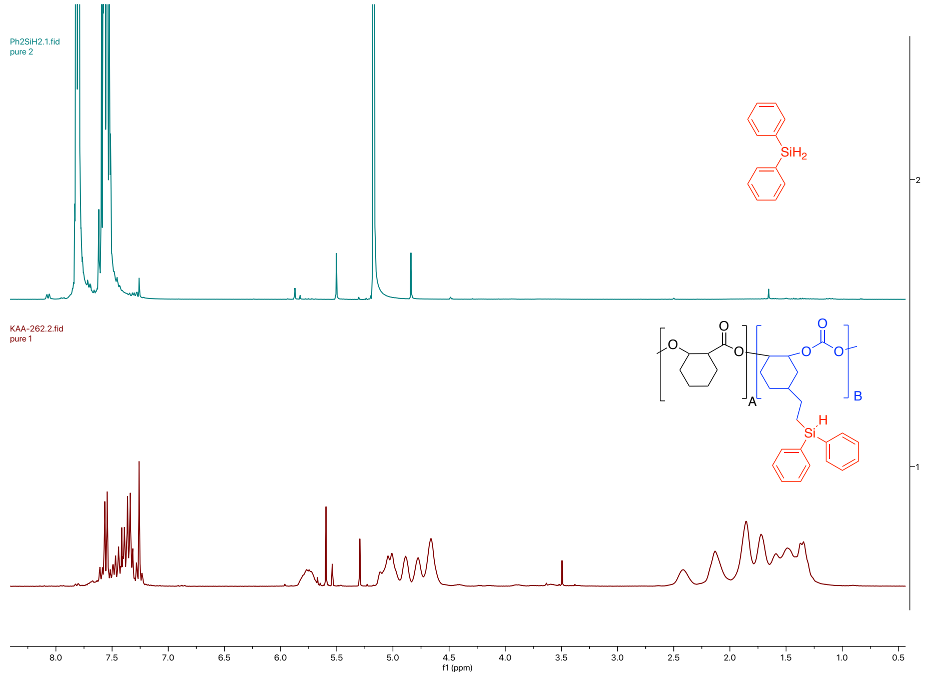

Figure S27. ^{1}H NMR (300 MHz, $CDCl_3$, 298 K) spectrum of pure Ph_2SiH_2 (top) and resulting functionalized terpolymer (CHO/VCHO/CO_2) (bottom). 36.4% of vinyl groups have been silylated (see Fig. S31 for expanded view).

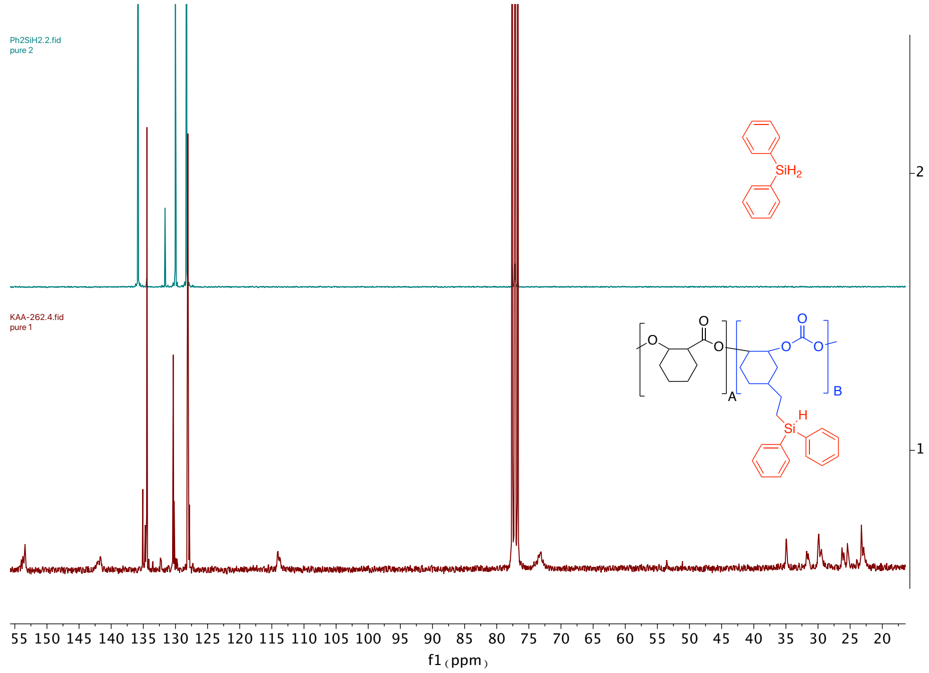

Figure S28. $^{13}C\{^{1}H\}$ NMR (125 MHz, $CDCl_3$, 298 K) spectrum of pure Ph_2SiH_2 (top) and resulting functionalized terpolymer (CHO/VCHO/CO_2) (bottom).

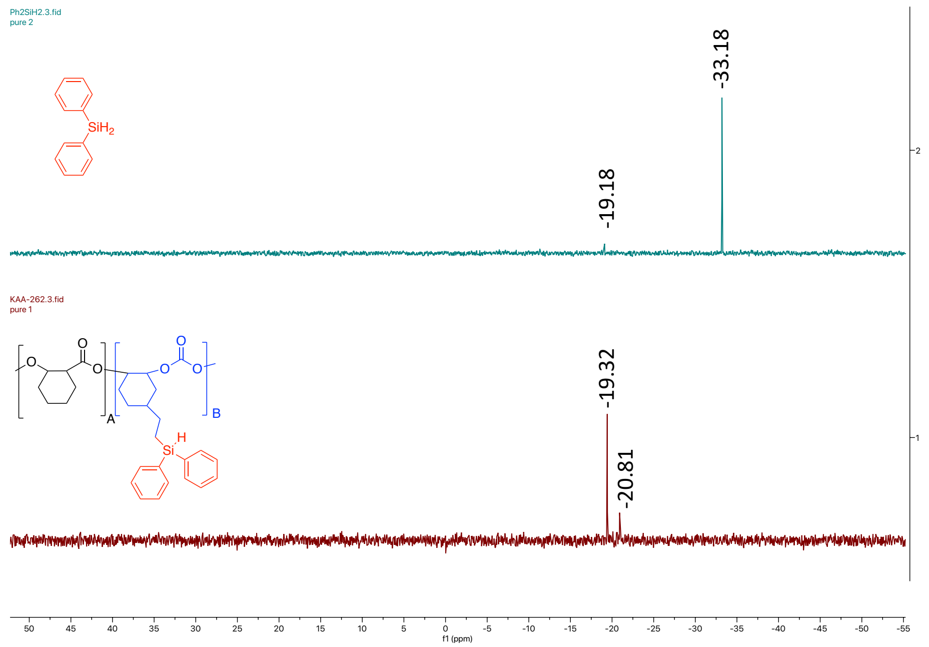

Figure S29. Refocused INEPT ^{29}Si NMR (60 MHz, $CDCl_3$, 298 K) spectrum of isolated PVCHC (top) and isolated silylated terpolymer (CHO/VCHO/CO_2) (bottom).

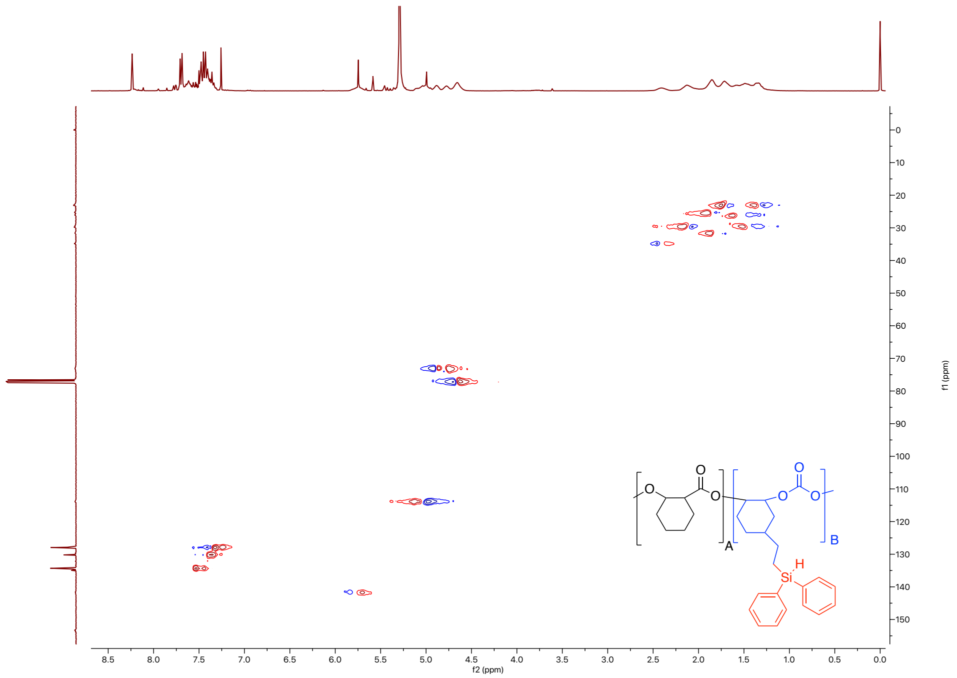

Figure S30. HSQC 2-D NMR spectrum of isolated silylated terpolmer (CHO/VCHO/CO_2). x-axis shows ^{1}H NMR spectrum (300 MHz, $CDCl_3$, 298 K) and y-axis shows ^{13}C{^{1}H} NMR spectrum (75 MHz, $CDCl_3$, 298 K) of the isolated polymer.

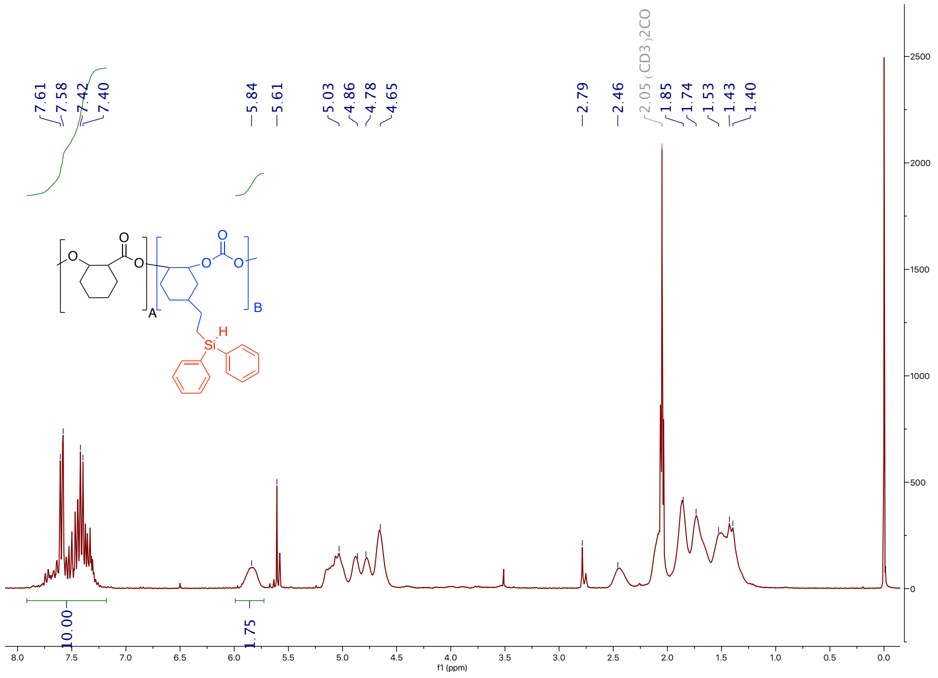

Figure S31. Integrated ^{1}H NMR (300 MHz, $(CD_3)_2CO$, 298 K) spectrum of silylated PVCHC. Normalized 1 Si*Ar*$_2$ group, 10H, which corresponds 1.75 C*H*$_{(B)}$ residual vinyl groups. % silyl = m/(m+n) *100% = 1/(1+1.75) *100% = 36.4% functionalization.

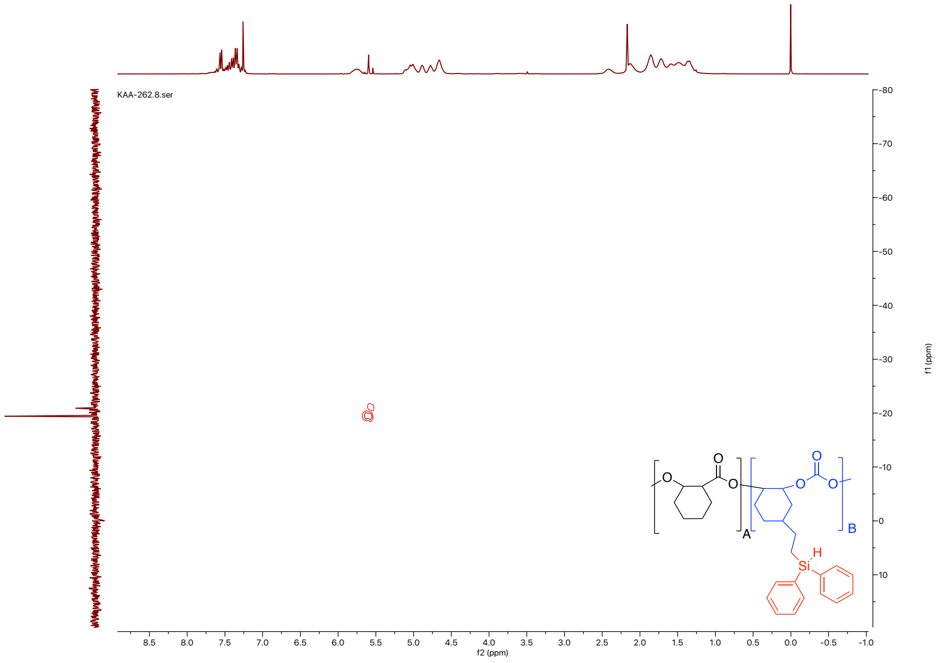

Figure S32. HMQC 2-D NMR spectrum of isolated silylated terpolmer (CHO/VCHO/CO_2). x-axis shows ^{1}H NMR spectrum (300 MHz, $CDCl_3$, 298 K) and y-axis shows ^{29}Si NMR spectrum (60 MHz, $CDCl_3$-*d*$_1$, 298 K) of the isolated polymer. J(H-Si)= 300 Hz

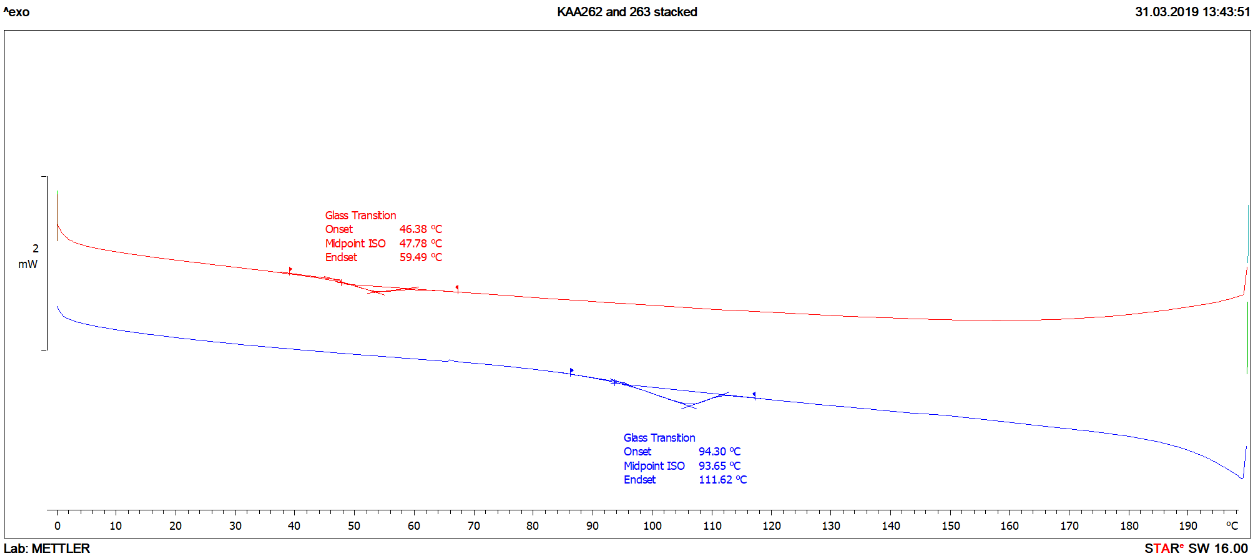

Figure S33. DSC of native terpolymer (CO_2/CHO/VCHO) from one pot reaction (blue) and after silane functionalization (red). Glass transition temperatures taken from the 3rd cycle.

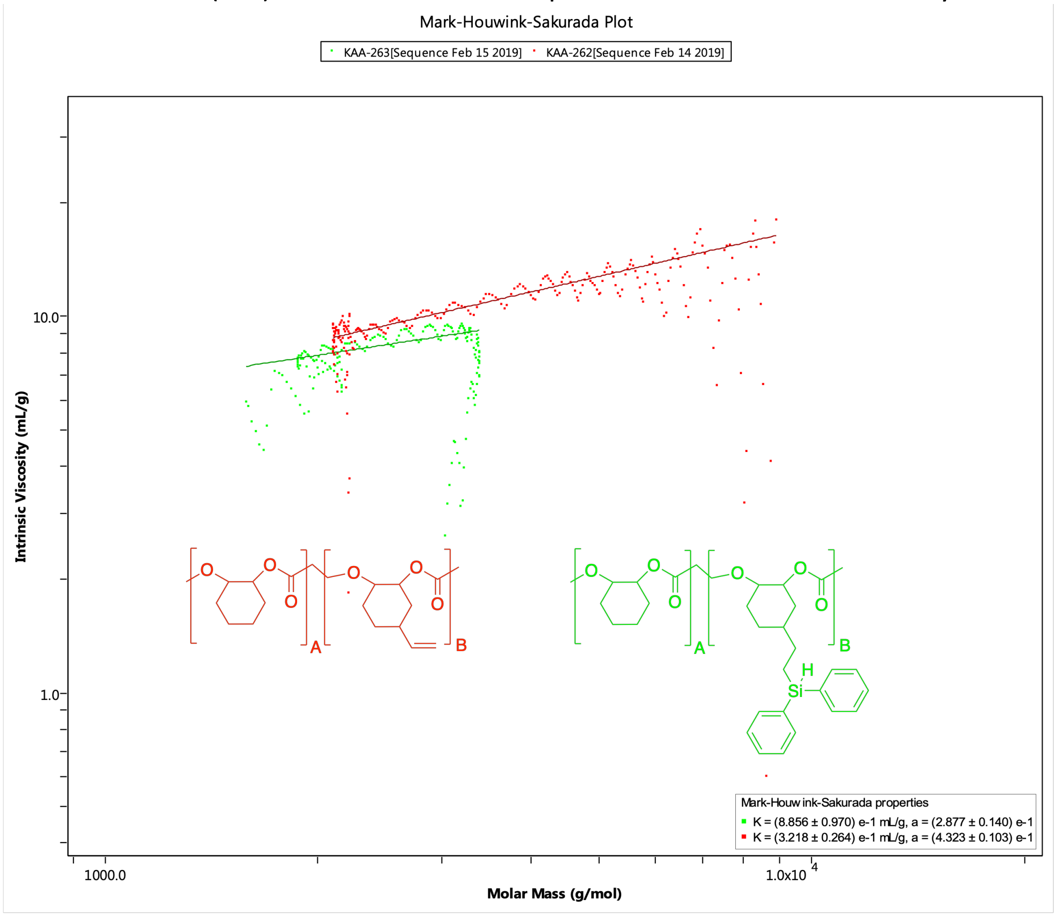

Figure S34. Mark-Houwink-Sakurada plot for isolated terpolymer (CHO/VCHO/CO_2) and the corresponding silylated terpolymer.

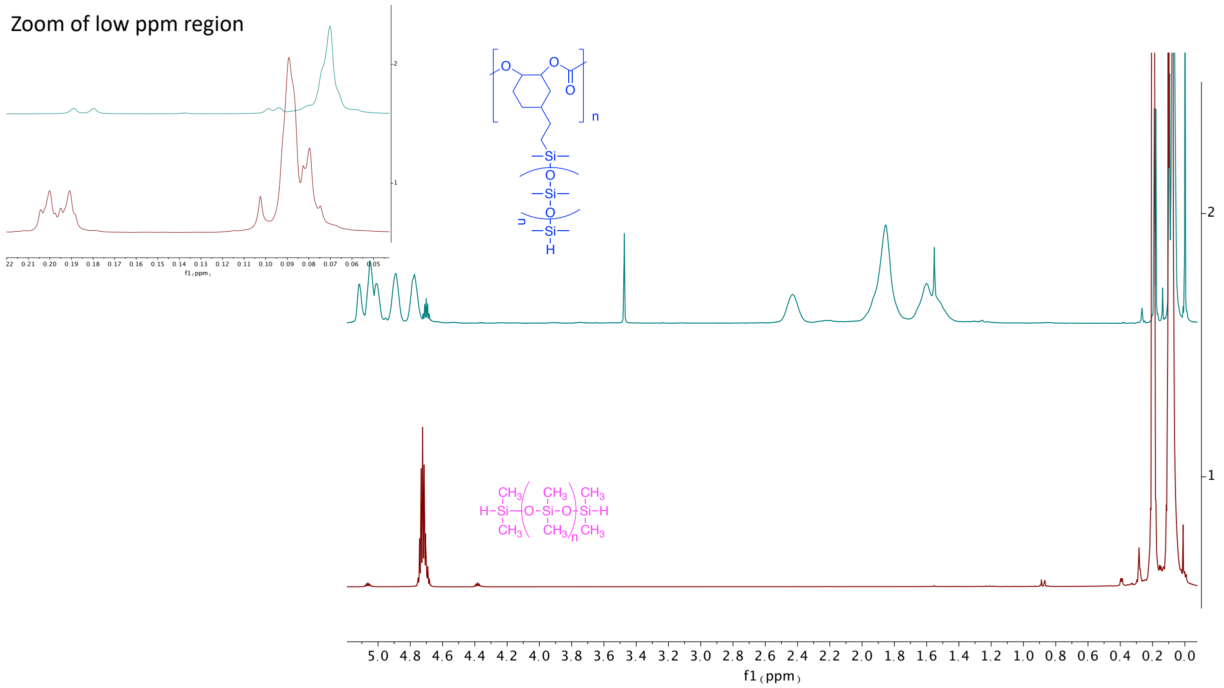

Figure S35. ^{1}H NMR (300 MHz, $CDCl_3$, 298 K) spectrum of pure DMS-HO3 (top) and resulting functionalized PVCHC (bottom).

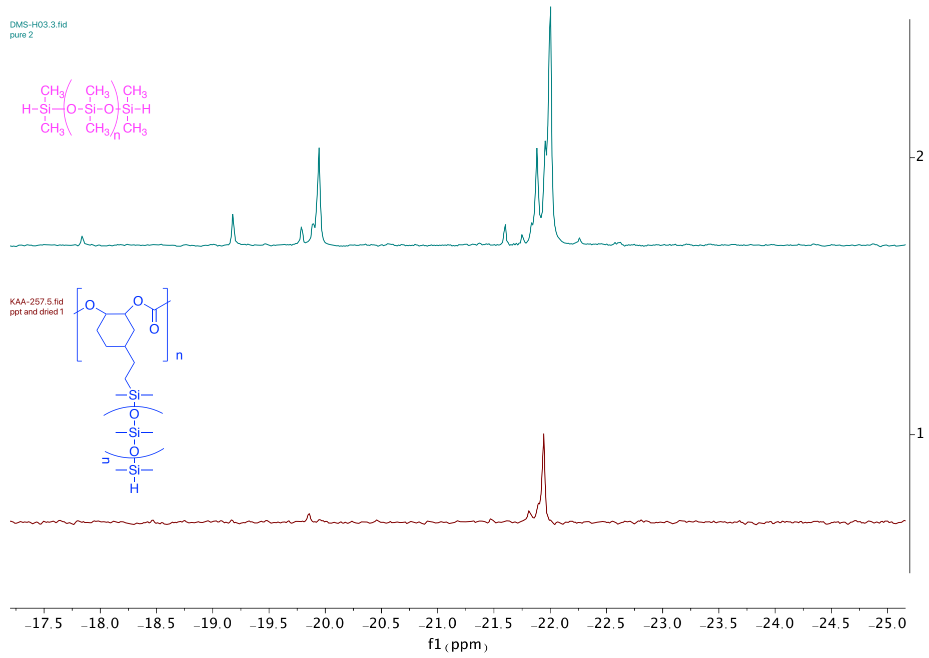

Figure S36. Refocused INEPT ^{29}Si NMR (60 MHz, $CDCl_3$, 298 K) spectrum of pure DMS-HO3 (top) and resulting functionalized PVCHC (bottom).

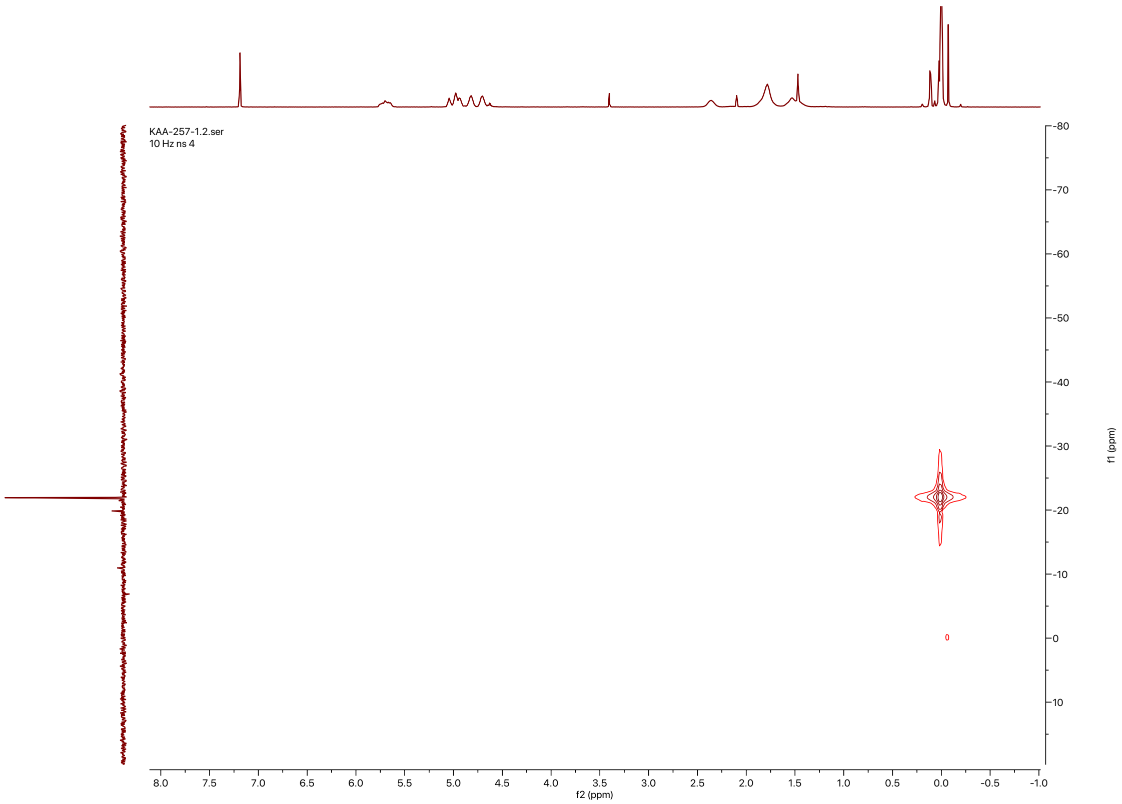

Figure S37. HMQC 2-D NMR spectrum of isolated DMS-HO3 functionalized PVCHC x-axis shows ^{1}H NMR spectrum (300 MHz, $CDCl_3$, 298 K) and y-axis shows ^{29}Si NMR spectrum (60 MHz, $CDCl_3$, 298 K) of the isolated polymer. J(H-Si)= 10 Hz

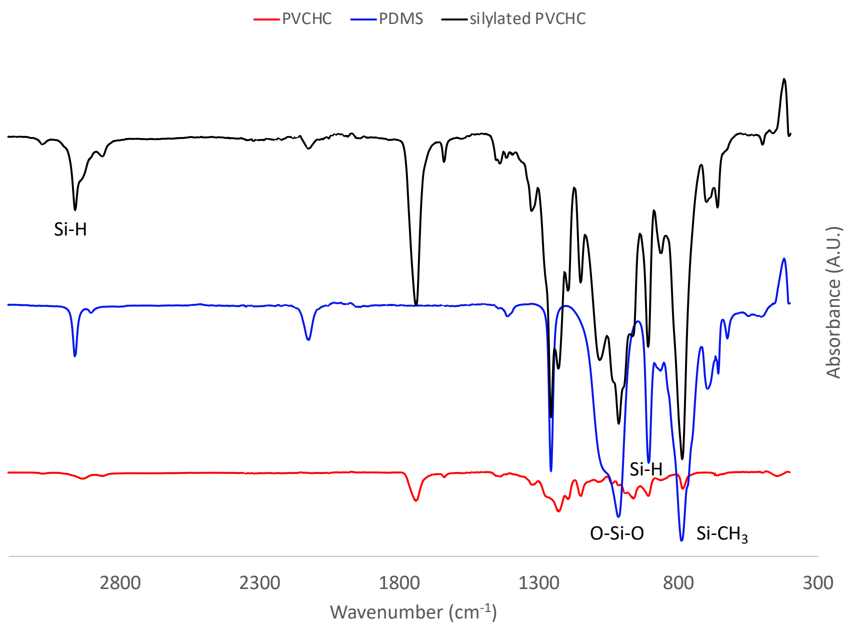

Figure S38. Stacked FTIR spectra of isolated PVCHC (red), DMS-HO3 (blue) and DMS-HO3 functionalized PVCHC (black)

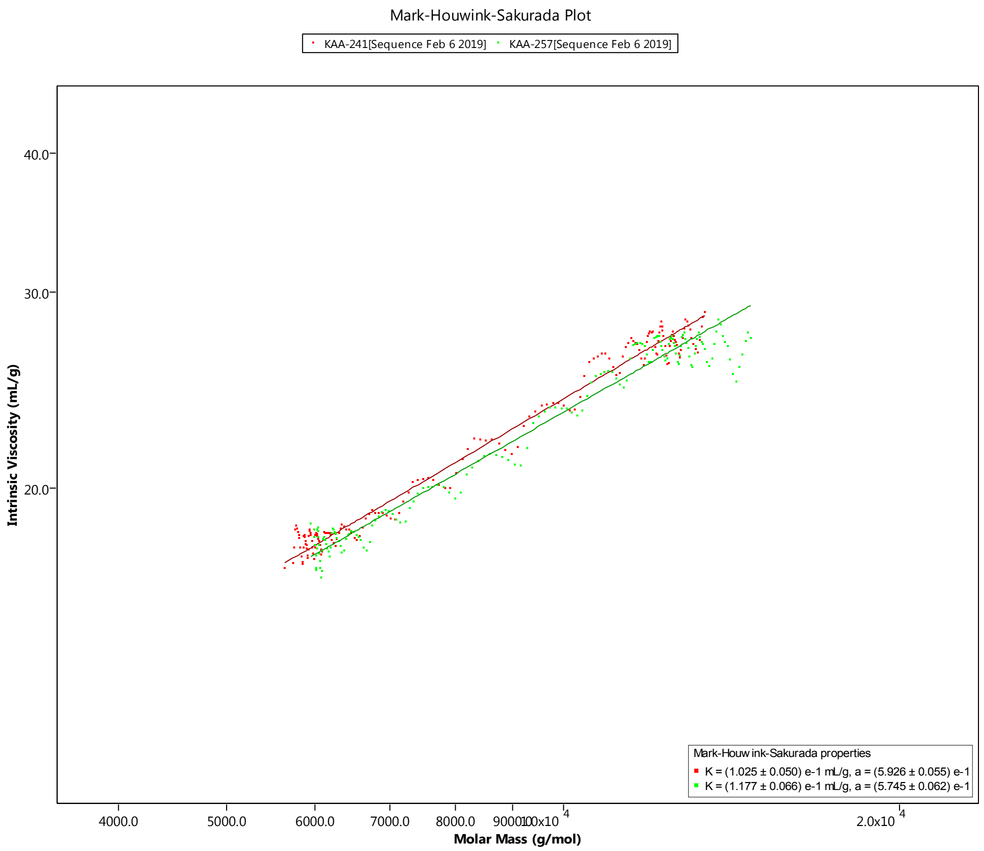

Figure S39. Mark-Houwink-Sakurada plot for isolated PVCHC (green) and DMS-HO3 functionalized PVCHC (red)
